# No evidence for differential sociosexual behavior and space use in the color morphs of the European common wall lizard (*Podarcis muralis*)

**DOI:** 10.1002/ece3.6659

**Published:** 2020-10-08

**Authors:** Javier Abalos, Guillem Pérez i de Lanuza, Alicia Bartolomé, Océane Liehrmann, Hanna Laakkonen, Fabien Aubret, Tobias Uller, Pau Carazo, Enrique Font

**Affiliations:** ^1^ Ethology Lab Instituto Cavanilles de Biodiversidad y Biología Evolutiva Universitat de València València Spain; ^2^ CIBIO/InBIO Centro de Investigação em Biodiversidade e Recursos Genéticos da Universidade do Porto Porto Portugal; ^3^ Ecology and Evolution Biology University of Turku Turku Finland; ^4^ Department of Biology Lund University Lund Sweden; ^5^ SETE Station d’Ecologie Théorique et Expérimentale UMR5321 Centre National de la Recherche Scientifique Paris France

**Keywords:** alternative strategies, color polymorphism, free‐ranging population, mesocosm, *Podarcis muralis*, social behavior

## Abstract

Explaining the evolutionary origin and maintenance of color polymorphisms is a major challenge in evolutionary biology. Such polymorphisms are commonly thought to reflect the existence of alternative behavioral or life‐history strategies under negative frequency‐dependent selection. The European common wall lizard *Podarcis muralis* exhibits a striking ventral color polymorphism that has been intensely studied and is often assumed to reflect alternative reproductive strategies, similar to the iconic “rock–paper–scissors” system described in the North American lizard *Uta stansburiana*. However, available studies so far have ignored central aspects in the behavioral ecology of this species that are crucial to assess the existence of alternative reproductive strategies. Here, we try to fill this gap by studying the social behavior, space use, and reproductive performance of lizards showing different color morphs, both in a free‐ranging population from the eastern Pyrenees and in ten experimental mesocosm enclosures. In the natural population, we found no differences between morphs in site fidelity, space use, or male–female spatial overlap. Likewise, color morph was irrelevant to sociosexual behavior, space use, and reproductive success within experimental enclosures. Our results contradict the commonly held hypothesis that *P. muralis* morphs reflect alternative behavioral strategies, and suggest that we should instead turn our attention to alternative functional explanations.

## Graphical abstract

Ventral color morphs in wall lizards have often been assumed to reflect alternative reproductive strategies. In this study, we study the social behavior, space use, and reproductive success of *Podarcis muralis* color morphs both in a free‐ranging population from the eastern Pyrenees and in ten experimental mesocosm enclosures. Overall, our results suggest that color morphs do not reflect alternative strategies concerning sociosexual behavior or space use.

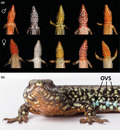

## 1 INTRODUCTION

Explaining the maintenance of phenotypic variability over time remains a central question in evolutionary biology. Population polymorphisms are a particularly widespread form of phenotypic variability (Galeotti, Rubolini, Dunn, & Fasola, 2003; Gray & McKinnon, 2007; Mckinnon & Pierotti, 2010; Roulin, 2004; Svensson, 2017). In polymorphic populations, individuals of the same sex and age may exhibit different phenotypes (e.g., color morphs) that are heritable, fixed in adults, and not condition‐dependent (Galeotti et al., 2003; Mckinnon & Pierotti, 2010; Roulin, 2004). Selectively neutral polymorphisms are expected to be lost eventually due to stochastic processes (i.e., genetic drift; Roulin, 2004), and the long‐term maintenance of polymorphisms within a population requires some form of balancing selection, for example, via nonrandom mating, source–sink dynamics, overdominance, or rare morph advantage (Galeotti et al., 2003; Roulin & Bize, 2007; Roulin, 2004; Svensson, 2017; Wellenreuther, Svensson, & Hansson, 2014).

Sexual selection often plays a major role in the maintenance of color polymorphisms (Roulin & Bize, 2007; Wellenreuther et al., 2014). Discrete variation among conspecifics in behavior or life histories associated with reproduction (termed alternative reproductive strategies, ARS) is frequently coupled with alternative color morphs (Ducrest, Keller, & Roulin, 2008; Roulin & Bize, 2007; Roulin, 2004; Shuster & Wade, 2003; Wellenreuther et al., 2014; Willink, Duryea, & Svensson, 2019; Zamudio & Sinervo, 2000). ARS are particularly frequent in males of polygynous (or polygynandrous) species, which experience a high variance in mating success and, thus, stronger sexual selection. In these species, the uneven distribution of fertilizations among males playing the conventional strategy allows the evolution of behavioral ARS (e.g., monogynist, satellite, sneaker) adapted to exploit distinct mating niches (Greenfield & Shelly, 2008; Shuster, 2008; Shuster, Briggs, & Dennis, 2013; Shuster & Wade, 2003; Taborsky, Oliveira, & Brockmann, 2008; Waltz, 1982). Genetically fixed strategies are favored whenever males tend to experience only one selective regime during their lifetime, so that specializing in alternative resources has higher fitness than being a generalist (Brockmann, 2001; Roulin, 2004; Zamudio & Sinervo, 2003). For instance, certain characteristic of the environment (e.g., heterogeneous distribution of resources, short breeding season) can interact with aspects of the species' ecology (e.g., short life span, adaptive site fidelity) producing resource‐defense mating systems (i.e., territoriality) in which subordinate males are unlikely to disperse. Males of such species tend to experience a single social environment during their lifetime, promoting the evolution of fixed, rather than conditional, behavioral strategies (Shuster & Wade, 2003; Zamudio & Sinervo, 2003). Balancing selection can maintain these alternative strategies, even if genetically fixed, whenever they obtain equal average fitness across contexts. This can happen in a wide array of scenarios, such as marked seasonality or spatial environmental heterogeneity (Brockmann, 2001; Taborsky & Brockmann, 2010). In sympatry, ARS can obtain equal fitness through frequency‐dependent selection (Gross, 1996; Shuster & Wade, 2003; Taborsky et al., 2008). Occasionally, two or more strategies can cycle in frequency over time if presenting a lower frequency confers a fitness advantage (negative frequency‐dependent selection (NFDS; Brockmann, 2001; Roulin, 2004; Taborsky et al., 2008; Takahashi, Yoshimura, Morita, & Watanabe, 2010; Willink et al., 2019). Color polymorphism may participate of this evolutionary process and be maintained under two different conditions. On the one hand, alternative color morphs may be directly selected for because of an adaptive advantage they confer in the context of ARS (e.g., sexual mimicry in damselflies; Svensson, Willink, Duryea, & Lancaster, 2020; Willink et al., 2019). Alternatively, color morphs may be an indirect by‐product of selection on other attributes related to the ARS (i.e., when genes involved in morphology, physiology, or behavior have pleiotropic effects on color production; Galeotti et al., 2003; Roulin & Bize, 2007; Roulin, 2004, 2016; Wellenreuther et al., 2014).

One of the best‐studied cases of color polymorphic ARS is the side‐blotched lizard, *Uta stansburiana*. Adult males of this species present one of three alternative throat colors (blue, orange, and yellow), each of which is associated with different sociospatial behaviors. Orange‐throated males establish large territories overlapping with several females by outcompeting blue‐throated males in territorial disputes. These vast territories make orange males vulnerable to losing fertilizations in favor of the nonterritorial yellow morph, which uses female mimicry to sneak copulations opportunistically. In turn, blue‐throated males compensate their competitive disadvantage by guarding females directly and hence securing more fertilizations against the yellow sneaker males (Alonzo & Sinervo, 2001; Calsbeek & Sinervo, 2002a; Sinervo & Lively, 1996; Sinervo et al., 2006; 2007; Sinervo & Zamudio, 2001; Zamudio & Sinervo, 2000;). This dynamic gives rise to periodic oscillations in the relative frequencies of *U. stansburiana* male color morphs, in a cyclical “rock–paper–scissors” (RPS) game whereby each color morph, when predominant, is vulnerable to invasion by another color morph (Sinervo & Calsbeek, 2006; Sinervo & Lively, 1996). These results sparked a proliferation of studies aimed at detecting similar differences in reproductive behavior among the numerous species of lizards with color polymorphism (Bastiaans, Morinaga, Castañeda Gaytán, Marshall, & Sinervo, 2013; Fernández et al., 2018; Huyghe, Herrel, Adriaens, Tadić, & Van damme, 2009; Huyghe, Vanhooydonck, Herrel, Tadic, & Van Damme, 2007; Olsson, Healey, & Astheimer, 2007; Olsson, Stuart‐Fox, & Ballen, 2013; San‐Jose, Peñalver‐Alcázar, Milá, Gonzalez‐Jimena, & Fitze, 2014; Yewers, Pryke, & Stuart‐Fox, 2016; Yewers, Stuart‐Fox, & Mclean, 2018). For a number of reasons, morph‐specific ARS, morph fluctuations, and rock–paper–scissors dynamics similar to those described in *Uta stansburiana* have been predicted to occur in Eurasian lacertids, particularly in wall lizards (genus *Podarcis*, family Lacertidae; Sinervo et al., 2007; Calsbeek, Hasselquist, & Clobert, 2010; Mangiacotti et al., 2019). First, ventral color polymorphisms involving three alternative colors (i.e., orange, white, and yellow) have been documented in adult individuals of at least 11 out of the 24 species currently recognized within the *Podarcis* genus, and is thus thought to have an ancestral origin (Andrade et al., 2019; Speybroeck, Beukema, Bok, Van der Voort, Velikov, 2016; Huyghe et al., 2007; Jamie & Meier, 2020; Pérez i de Lanuza, Bellati, Pellitteri‐Rosa, Font, & Carretero, 2019; Runemark, Hansson, Pafilis, Valakos, & Svensson, 2010; Sacchi et al., 2007). Second, many of these species show high site fidelity, low interannual survival, and occupy habitats where resources relevant to reproduction (e.g., stone walls) are unevenly distributed (Barbault & Mou, 1988; Calsbeek et al., 2010; Carretero, 2007; Edsman, 1990, 2001; Font, Barbosa, Sampedro, & Carazo, 2012; Sinervo et al., 2007; Strijbosch, Bonnemayer, & Dietvorst, 1980). Third, males of many wall lizards experience strong intrasexual competition, mainly in the contexts of territorial disputes and sperm competition. Females seem to be attracted to high‐quality and/or familiar patches of habitat rather than to males with certain phenotypic characteristics (Edsman, 1990, 2001; Font, Barbosa, et al., 2012). Moreover, behavioral observations and genetic analyses have confirmed that receptive females often mate with more than one male before oviposition, which results in a high incidence of multiple paternity (Heathcote et al., 2016; Oppliger, Degen, John‐Alder, & Bouteiller‐Reuter, 2007; Uller & Olsson, 2008). Consequently, adult males try to secure fertilizations by investing significant time and energy in the defense of territories offering resources valuable to females (such as basking spots, shelters, optimal egg‐laying sites) against other males (Baird, 2013; Edsman, 1990; Font, Barbosa, et al., 2012). The outcome of this territorial disputes is crucial to male reproductive success, and patterns of shared paternity have often been found to reflect spatial and social dominance among males (MacGregor, Lewandowsky, et al., 2017; MacGregor, While, et al., 2017; Oppliger et al., 2007; Uller & Olsson, 2008; While et al., 2015). For these reasons, alternative color morphs in many wall lizards are often believed to represent the visible mark of heritable ARS involving differential sociospatial behaviors in males (Andrade et al., 2019; Calsbeek et al., 2010; Huyghe et al., 2007; Pérez i de Lanuza, Carretero, & Font, 2017; Sinervo et al., 2007).

**Figure 1:**
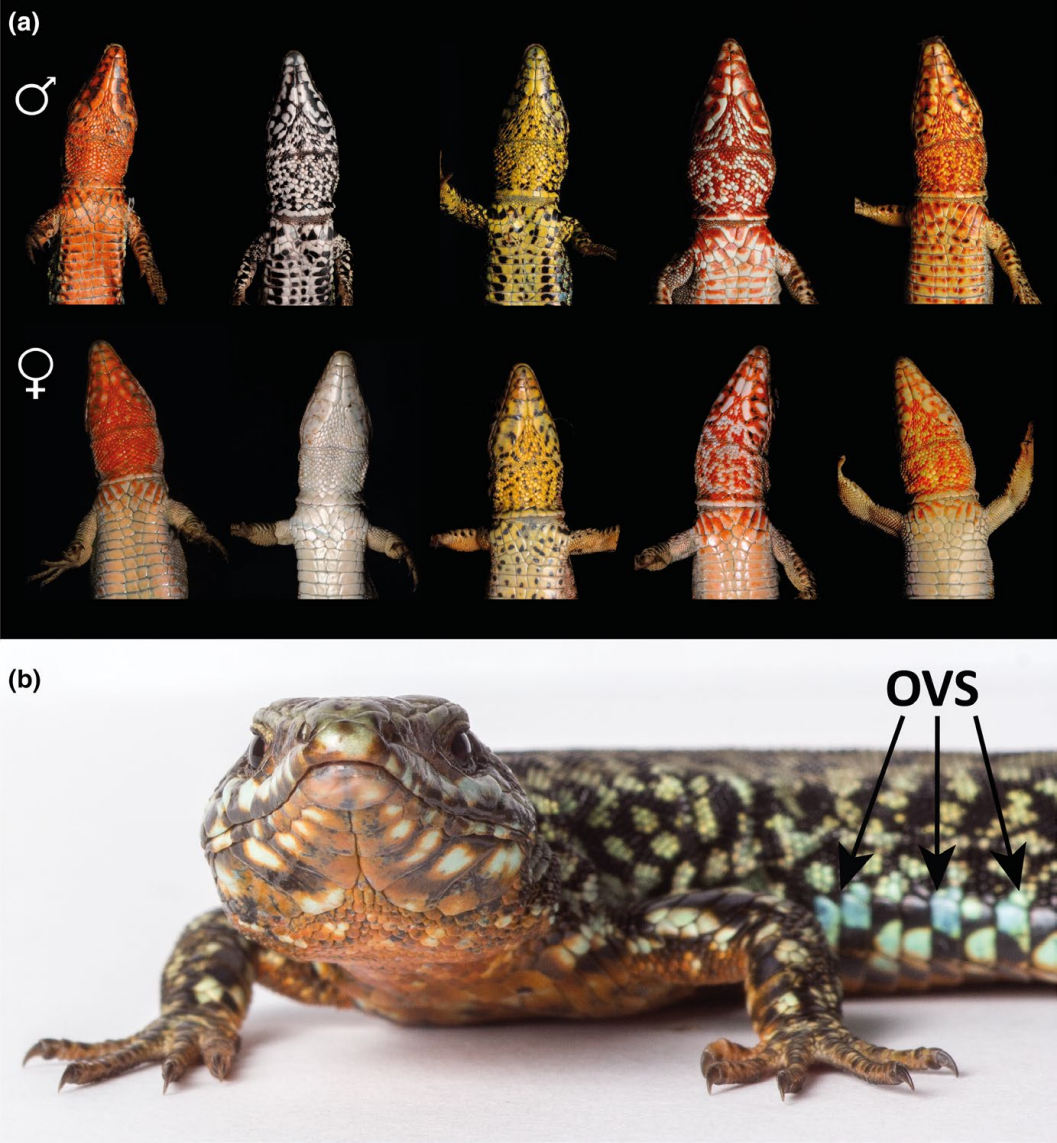
(a) Color variation in the ventral surface of adult *Podarcis muralis* lizards. (b) Close‐up of an orange morph male showing UV‐blue and black spots in its outer ventral scales (OVS)

The European common wall lizard (*Podarcis muralis*) shows the widest distribution within the genus *Podarcis*, and many populations exhibit a striking color polymorphism (Speybroeck, Beukema, Bok, Van der Voort, and Velikov, 2016). Adults of both sexes may show up to five alternative ventral color morphs: three uniform (pure) morphs, that is, orange (O), white (W), and yellow (Y), and two intermediate mosaics combining orange and white (OW) or yellow and orange (YO) (Pérez i de Lanuza, Font, & Carazo, 2013; 2019; Figure 1). These color morphs are fixed at maturity (Pérez i de Lanuza et al., 2013), and recent research suggests that orange and yellow color expression is caused by recessive homozygosity at two separate loci in the regulatory regions of two genes associated with pterin (SPR) and carotenoid (BCO2) metabolism, respectively (Andrade et al., 2019). Interestingly, each of these morphs is found in geographically distant sublineages of the species thought to have diverged up to 2.5 million years ago (Andrade et al., 2019; Salvi, Harris, Kaliontzopoulou, Carretero, & Pinho, 2013; Figure S1). Local morph composition shows considerable geographical variation, although white ventral coloration is typically the most common (>50%), while the orange and especially the yellow morph rarely predominate. The yellow and yellow‐orange morphs are often the most infrequent, and in Pyrenean populations, they seem to be geographically restricted to a subset of localities (<50%) characterized by male‐biased sex ratios and marked climatic seasonality (Pérez i de Lanuza et al., 2017; Pérez i de Lanuza, Sillero, & Carretero, 2018).

At least the pure morphs in *P. muralis* are often assumed to reflect alternative behavioral or life‐history strategies (e.g., Calsbeek et al., 2010; Galeotti et al., 2010; Scali et al., 2013; Zajitschek, Zajitschek, Miles, & Clobert, 2012). The colors are indeed well suited to function as color signals. They are highly conspicuous to the species visual system and heritable, and their ventral position allows the lizards to control their exposure through posture (Andrade et al., 2019; Pérez i de Lanuza, Carretero, & Font, 2016; Pérez i de Lanuza & Font, 2015, 2016). Moreover, the alternative colors show discrete variation and are chromatically discriminated as categorically distinct by conspecifics (Pérez i de Lanuza, Ábalos, Bartolomé, & Font, 2018; Pérez i de Lanuza et al., 2013), which makes them particularly suited to convey information about strategy (Tibbetts, Mullen, & Dale, 2017). Research on *P. muralis* has revealed several differences in morphological, physiological, and behavioral traits across color morphs (e.g., Calsbeek et al., 2010; Galeotti et al., 2013; Pérez i de Lanuza & Carretero, 2018; Sacchi, Mangiacotti, Scali, Ghitti, & Zuffi, 2017; Scali et al., 2013; Zajitschek et al., 2012). However, there is no clear evidence that these correlated traits reflect morph‐specific strategies, whether in the context of sexual or natural selection. Furthermore, available studies have focused on morphology and physiology (Calsbeek et al., 2010; Galeotti et al., 2007, 2010; Galeotti, 2013; Pellitteri‐Rosa, 2010; Sacchi, Mangiacotti, et al., 2017; Sacchi et al., 2007), while central aspects in the behavioral ecology of this species have received little attention (Abalos, Pérez i de Lanuza, Carazo, & Font, 2016; Pellitteri‐Rosa et al., 2017; Sacchi et al., 2015; Sacchi et al., 2009). In particular, the interaction between sociospatial behavior, reproductive success, and shared paternity is key to ascertain whether *P. muralis* color morphs obtain their fitness using alternative behavioral strategies during the breeding season. If behavioral ARS underlie color polymorphism in *P. muralis*, the alternative color morphs may show equal reproductive success but differential investment in social dominance, territoriality, space use, and/or postcopulatory sexual behavior (e.g., mate‐guarding), which often translate into morph‐biased patterns of cosiring and clutch monopolization (Formica, Gonser, Ramsay, & Tuttle, 2004; Sinervo & Lively, 1996; Sinervo, Miles, Frankino, Klukowski, & DeNardo, 2000; Zamudio & Sinervo, 2000). However, no previous study has investigated the alignment of polymorphic coloration, social behavior, and reproductive performance in sufficient detail to draw firm conclusions about the existence of behavioral ARS in *P. muralis*. To fill this gap, we monitored morph differences in spatial behavior in a free‐ranging polymorphic population from the eastern Pyrenees across a period of 5 years. We complemented this with a mesocosm experiment using ten experimental populations with balanced sex ratio and morph frequencies to study the spatial and sociosexual behavior of *P. muralis* pure color morphs in a controlled environment. Our experimental design was aimed to detect behavioral differences in space use or social behavior among the color morphs, as well as morph differences in shared paternity, rather than frequency‐dependent effects on morph fitness. For this reason, we introduced the morphs in equal frequencies to optimize our sample size of individual lizards representing each morph within the enclosures. Incidentally, as the balanced morph ratios employed are highly unlikely to occur in natural populations, this design also allows us to test whether the higher prevalence of white morph lizards observed across the species distribution range results from some form of frequency‐dependent fitness effect.

## 2 MATERIALS AND METHODS

### 2.1 **Spatial behavior in a free‐ranging population of** *Podarcis muralis*

**Figure 2:**
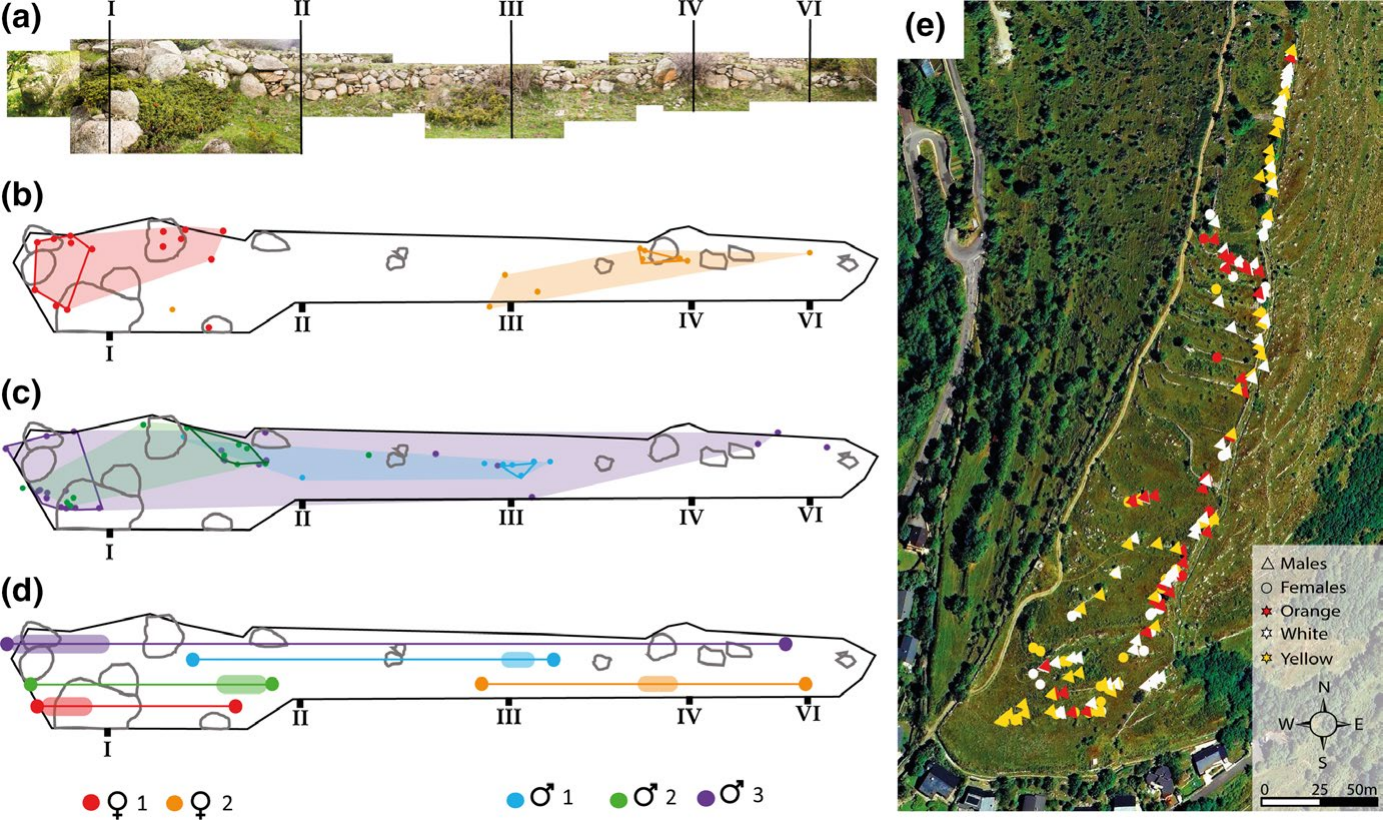
Space use in a free‐ranging population of *P. muralis*. (a) Photographic composition of a stone wall in Angoustrine. Roman numbers mark reference points for precision. (b and c) Schematic representations of the wall vertical surface used as home‐ (color shades, 95% MCP) and core ranges (solid‐line polygons, 50% MCP) by two females (b) and three males (c) during the breeding season of 2010. (d) Diagram of the linear home‐ and core range lengths estimated for each lizard as the width of the corresponding MCP (solid‐lines = home range, color shades = core range). (e) Google Earth satellite image of the study site in Angoustrine (Map data: Institut Cartogràfic de Catalunya), with arithmetic center of each pure morph lizard core range during the period examined (367 lizards, 125 females, and 242 males)

During the spring seasons of 2006–2010, we collected data on the activity and spatial behavior of a population of wall lizards in Angoustrine (42°28′43″N, 1°57′12″E), eastern Pyrenees. The study site (ca 140 × 500 m = 7 ha; Figure 2) consists of a series of abandoned terraced fields characterized by granite outcrops and old dry‐stone walls partially covered in vegetation (see Font, Barbosa, et al., 2012). Lizards were mostly sighted perching on the stone walls, usually remaining within the boundaries of a single wall for the whole breeding season. In any particular year, lizards showing at least six resightings on the same wall were considered resident, while lizards showing five or fewer resightings and/or sighted at walls located more than 100 m apart were considered nonresident transients (Edsman, 1990). We only considered lizards measuring at least 56 mm from snout to vent (SVL), which ensures they had developed full‐blown adult ventral coloration (Figure S2; Pérez i de Lanuza et al., 2013).

To examine potential intermorph differences in activity levels, for each lizard we counted the total number of sightings, the mean days elapsed between consecutive sightings, and the mean distance between consecutive sightings. As frequently done when a species' habitat is physically constrained (e.g., river fauna; Ahlers, Heske, Schooley, & Mitchell, 2010; Kornilev, Dodd, & Johnston, 2010; Kramer, 1995), we calculated a one‐dimensional measure of home range size for each lizard inhabiting a particular stone wall. We operationally defined the width of the 95% minimum convex polygon (MCP) encompassing the lizard's cluster of resightings on a stone wall as the lizard's linear home range size, and the width of the 50% MCP as the linear core range in which the animals were most frequently observed (Figure 2; Grassman, Tewes, Silvy, & Kreetiyutanont, 2005; Heupel, Simpfendorfer, & Hueter, 2004; Powell, 2000). To determine what fraction of male core ranges constitutes a territory (Maher & Lott, 1995), we defined the exclusive range of each male as the part of its core range that did not show overlap with the core range of any other male (i.e., territory; Kerr & Bull, 2006). Then, for each male with a reliable linear home range estimate (≥17 sightings; see Appendix S1) we measured spatial overlap by counting the number of resident females whose core ranges overlapped at least partially with either the home range, or the exclusive range of the focal male. To account for the vertical dimension of the lizards' home ranges, we also calculated the mean perching height of each resident lizard sighted.

### 2.2 Mesocosm experiment

#### 2.2.1 Lizard capture and housing

We captured 190 lizards (100 females and 90 males) by noosing from 12 polymorphic localities spread across the Cerdanya Valley (Eastern Pyrenees). In each of these localities, we captured 2–8 lizards (SVL ≥ 56 mm) showing each of the pure color morphs (O, W, Y) so as to avoid a geographical bias in our sample. No lizards were captured from populations lacking any of the pure color morphs. To ensure captured females were not gravid, we captured females at the end of the previous breeding season (September 2017), and transferred them to the Statión d’Ecologie Théorique et Expérimentale (SETE, Moulis, France). There, we housed females in groups of 3–5 coming from the same locality in outdoor circular plastic tanks (170 cm diameter, 60 cm high), where they were kept under natural conditions for 130 days (Bestion, Teyssier, Aubret, Clobert, & Cote, 2014; Le Galliard, Ferriere, & Clobert, 2005). In May 2018, after an artificial hibernation period (see Appendix S1), we reinstalled the females in the outdoor tanks for 2 weeks while we captured the males.

#### 2.2.2 Morphometry

Two days before the onset of the experiment, we measured SVL (0.1 mm) and mass (±0.01 g) of each lizard with a ruler and a spring balance (Pesola, Schindellegi, Switzerland). Using a digital caliper (±0.01 mm; Mitutoyo, Telford, UK), we quantified interlimb length (ILL) in females, and two head measurements in males: length (HL) and width (HW) (Olsson, Shine, Wapstra, Ujvari, & Madsen, 2002). We also removed ~5 mm from the tail tip of each individual and preserved the tissue in 90% ethanol for genetic analyses.

#### 2.2.3 Experimental enclosures and egg incubation

To study social behavior and mating patterns in ten experimental populations of *P. muralis*, we released 180 lizards of either sex into ten experimental enclosures at the Metatron research facility (Caumont, France; Legrand et al., 2012). Within each of these enclosures, we created two types of sites that varied in structural complexity. Each site consisted of a wooden pallet (~1.2 m^2^) with differing number of bricks, cinderblocks, rocks, and logs piled above, which acted both as shelter and as basking sites (Figure S3.). We arranged high‐ and low‐quality sites (respectively HQ and LQ) in two rows of three pallets along the N‐S axis, separated by a line of six rocks (which we also considered as LQ habitat) (MacGregor & While, et al., 2017). We then surrounded the area with a plastic barrier (70 cm high) to prevent any escapes or intrusions. In total, each experimental cell had 47 m^2^.

On 23 May 2018, we released nine males (3O:3W:3Y) within each of the enclosures (simultaneously and always from the southeast corner). We monitored male behavior (see below) for 7 days before releasing nine females (3O:3W:3Y) within each enclosure. Due to posthibernation mortality, the white female morph was underrepresented in two of the ten experimental enclosures (5o:1w:3y). Prior to release, we marked each lizard permanently on the ventral scales using a disposable medical cautery unit (Ekner, Sajkowska, Dudek, & Tryjanowski, 2011) and drew a dorsal number with a toluene xylene‐free permanent marker to facilitate individual recognition during behavioral observations (see Video S1 in the Appendix S1; Ferner & Plummer, 2016). To minimize the noise introduced by size asymmetries and prior social interactions, we allowed a maximum SVL difference of 2 mm (within‐sexes) and only put lizards together in the same experimental enclosure if they had been captured at least 300 m apart.

On 22 June, we released the males at their capture location (previously determined using a GPS device) and housed females individually in the laboratory until oviposition (see Appendix S1). We lost 22 clutches due to females laying eggs before we retrieved them from the enclosures (12 females) or because they failed to produce a clutch (10 females). These lost clutches were evenly distributed across enclosures (*χ^2^* = 14.667, *p* = .10) and female morphs (orange = 8, white = 8, yellow = 6). For the remaining 68 females, we counted the number of fertile and infertile eggs within each clutch by noting the presence of a calcified shell and vascularization 48 hr after oviposition (Köhler, 2006). We incubated the resulting 230 fertile eggs in plastic cups filled with moist coco husk (1:2 coco:water by weight) and covered with a perforated lid at a constant temperature of 28°C (Memmert GmbH + Co.KG incubator, Schwabach, Germany). Upon hatching, each of the 209 born juveniles was measured (SVL), weighted, sampled for DNA, permanently marked, and released at the outdoor tanks in the SETE Moulis. For 21 embryos that died before hatching, we obtained DNA samples via dissection of the eggs. Average clutch size was 5.57 ± 0.20 eggs, average fertilization success (fertile eggs/clutch size) was 67%, and average hatching success (hatched/fertilized eggs) was 90%.

#### 2.2.4 Behavioral observations

From 23 May to 22 June, we conducted observations of spatial and social behavior at the natural peak activity hours for the lizards (9.30–14.30; 16.30–19.30), spacing consecutive visits to the same enclosure at least 1 hr and ensuring an even distribution of observations across the different time periods. Two researchers (JA and AB) recorded the identity, position, and behaviors of the lizards participating in social interactions using a behavior sampling rule in recording sessions lasting 40 min. A social interaction was considered to occur whenever a marked lizard in our visual range directed any of the behaviors listed in Table 1 toward a conspecific. During interactions, we recorded the first occurrence of the behaviors performed by each lizard. Consecutive interactions involving the same lizards were recorded as different events whenever the participants remained further than 30 cm apart for longer than 2 min. To ensure interobserver reliability, JA and AB collected behavioral data together for the first 6 days of the experiment (Cohen's *kappa ± *CI_95%_ = 0.87 ± 0.05; Kaufman & Rosenthal, 2009). A third observer (OL) performed sequential rounds visiting all the enclosures every 2.5 hr to collect data on the lizards' spatial behavior. Using scan sampling, we determined the identity and location of every lizard in sight on a scale map of the enclosure that included the six wooden pallets. Each enclosure was observed from a starting position located 1 m from the plastic barrier surrounding it for 5 min, and then walking around it (randomizing direction between consecutive visits) to record lizards that were not visible from the starting position. To balance sampling effort across enclosures, scanning of a single enclosure was restricted to a maximum period of 15 min after the first lizard was spotted.

**Table 1:** Partial ethogram used during behavioral observations to collect data on social interactions within the experimental enclosures

Behavior	Description
Approach^a^	Movement toward a nonfleeing conspecific
Display	Gular extension, back‐arching, shoulders raised, head down, sagittal compression (any combination)
Bite	One or more bites to another individual (excluding tail grab)
Retreat^a^	Movement away from a nonchasing conspecific
Chase	Rapidly following another FLEEING lizard
Flight	Fast‐paced movement to withdraw from a CHASING lizard
Foot shakes II^b^	Sequence of front‐leg waves in the air or onto the substrate
Tail grab	A male bites the tail or inguinal region of a female. Often followed by copulation
Tail shake	Shaking entire tail (or its posterior portion) swiftly from side to side
Mating	Two lizards engage in copulation
Coperching	Two or more lizards lying together in close vicinity (<15 cm; >30 s)
Cloacal drag	Pulling body forward while keeping cloaca in contact with substrate

^a^ We classified the mode of locomotion used as either running (fast‐paced) or any other mode of locomotion (slow‐paced).

^b^ *Podarcis muralis* lizards perform four types of foot shake displays (named I, IIa, IIb, and III; see Font et al., 2012 and references therein), of which two (IIa and IIb) are given in a social context. We only recorded these two types of foot shakes. Type IIa: rapid large amplitude vertical movements of front legs frequently performed by females in male–female interactions (belly‐down, head‐up posture). Losers of male–male agonistic interactions often perform this type of foot shakes, which are hence considered in this context as submissive/appeasement displays (see Font & Desfilis, 2002; Aragón, López, & Martín, 2006 for details in other *Podarcis* lizards). Type IIb: Performed by males when approaching females (limbs extended, often displaying; Pérez i de Lanuza, Font, et al., 2016).

#### 2.2.5 Behavioral analyses

We classified the interactions according to their sociosexual context into four types: intrasexual competitive and noncompetitive, and male–female reproductive and nonreproductive. Intrasexual interactions were deemed competitive whenever one lizard (i.e., the loser) used fast‐paced locomotion to flee from another lizard (i.e., the winner) showing display behavior and/or physical aggression (i.e., display, bite, or chase). In males, where competitive encounters were numerous, we used the R package BradleyTerry2 to fit a Bradley–Terry model to the observed matrix of contest outcomes within each enclosure to obtain an individual index of social dominance for every male (further details in Abalos et al., 2016; Firth & Turner, 2012; Stuart‐Fox, Firth, Moussalli, & Whiting, 2006). To examine potential nontransitive relations of dominance among male color morphs, we also fitted three logistic mixed models (one for each morph) on the contest outcome of heteromorphic encounters and tested whether the probability of winning against other morphs differed from even odds. Male–female interactions were classified as reproductive when the lizards engaged in sex‐specific display behaviors (i.e., ♂: display; ♀: foot shakes, tail shake), copulatory behavior (i.e., tail grab, mating), or prolonged physical vicinity (i.e., coperching). To examine the effect of morph combination on the frequency of male–female reproductive interactions, we used social network analysis on the compiled version of SOCPROG (Whitehead, 2009) (Appendix S1).

Positional data were used to examine the putative effect of color morph on activity, space use and overlap with conspecifics. To account for habitat use within the enclosures, we estimated range areas by adjusting the smoothing factor in a fixed‐kernel contour analysis until it matched the area of the 95% MCP (smoothing multiplier = 0.75, matrix cell number = 40; Kie, 2013; Row & Blouin‐Demers, 2006; MacGregor, Lewandowsky, et al., 2017; MacGregor, While, et al., 2017). Lizards with fewer than nine sightings (*N = *3) were excluded from the analysis (see Appendix S1). For each lizard, we calculated range size and overlap with conspecifics both at the 95% (home range) and at the 50% (core range) isopleth levels. Each lizard was assigned to a high‐ or low‐quality site based on the position where the 50% kernel estimate indicated peak density. Because of the high lizard density within the enclosures, male‐exclusive areas were peripheral and uninformative, so we did not conduct further analyses on them. When calculating home range estimates, we excluded the positional data collected during the first 6 days of the experiment to allow for an acclimation period. All spatial analyses were conducted in Ranges 9 (Anatrack Ltd., UK; Kenward, Casey, Walls, & South, 2014).

#### 2.2.6 Parentage analyses

We isolated DNA from tail‐tip samples using the DNeasy 96 Blood & Tissue Kit (Qiagen, Valencia, CA, USA), obtaining a final elution volume of 150 µl in AE buffer. We then combined the primers of six microsatellite loci described in *P. muralis* (Heathcote, Dawson, & Uller, 2014; Richard et al., 2012) into two different multiplexes (MPA: Pm16, Pm09, PmurC168; MPB: Pm19, Pm14, PmurC038) and ran standard PCR with 26 cycles and a final extension step of 30 min at 60°C. Forward primers were labeled with different fluorescent dyes (FAM, NED, HEX). Diluted PCR products (1:5) were genotyped together with an internal ladder (Red ROX‐500) on an ABI 3130 genetic analyzer (Applied Biosystems Inc.). One researcher (HL) scored the alleles for every adult and juvenile lizard in Geneious 7.0.4 (Biomatters, available at http://www.geneious.com), which we used to conduct parentage analysis in Cervus 3.0 (Kalinowski, Taper, & Marshall, 2007; Marshall, Slate, Kruuk, & Pemberton, 1998). We assigned paternity based on the log‐likelihood statistic of each mother–father–offspring trio (LOD scores), using two confidence levels (strict: 95%, relaxed: 80%) and the nine males within each enclosure as candidate fathers. Critical LOD scores were determined by running a simulation paternity analysis based on 100,000 offspring with known mothers and nine candidate fathers. We could reliably assign paternity to every offspring examined (strict: 209 juveniles, relaxed: 229 juveniles).

To quantify individual fitness, we operationally defined two variables based on the results of the paternity analysis: mating success (i.e., the overall number of different mates with whom a lizard conceived offspring) and reproductive success (i.e., the total number of embryos/hatchlings sired). Since selection will depend on relative rather than absolute fitness, we then divided the fitness measures of each lizard by the mean for all same‐sex conspecific within its enclosure. In addition, to evaluate intermorph differences in sperm competition intensity, for each male we determined the average number of competitors with which he shared paternity of a clutch.

#### 2.2.7 Statistical analyses

We ran linear mixed models using the *lme4* package (Bates, 2014) in R (R Core Team, 2019), and model selection was conducted using backward single‐term deletions (*p* < .05) of the saturated model followed by model comparisons via likelihood‐ratio tests (at *α* = 0.05). All numerical variables were centered and scaled before running the models (Schielzeth, 2010). We checked that all response variables conformed to homoskedasticity and normality assumptions before assuming a Gaussian distribution in model fitting. For some variables that did not conform to these assumptions even after transformation, we fitted models using different distributions (Appendix S1).

#### 2.2.8 Power analysis

Using G*Power (Erdfelder, Faul, & Buchner, 1996) and the methodology provided by Thalheimer and Cook (2002), we determined the effect size for an array of published morph differences detected in *U. stansburiana* and other polymorphic lizards thought to present some form of ARS (Table S1). We then used G*Power to calculate the smallest effect size that our sample size from the free‐ranging population allowed us to detect (sensitivity analysis), and the sample size required to detect biologically meaningful differences among morphs in the mesocosm experiment (a priori required sample size). We chose the more conservative approach of conducting these a priori analyses in G*Power instead of by simulation since this latter approach requires the researcher to directly determine estimates for both fixed and random effects, for which we had no previous reliable information (Green & Macleod, 2016). However, to better accommodate for the mixed‐model statistical design of our experiments, we additionally used the estimates obtained here to run a simulation‐based analysis of power on the probability of detecting medium‐sized (Cohen's *d* > 0.5) and large (Cohen's *d* > 0.8) effects with growing sample sizes (Haenlein & Kaplan, 2004; Hoenig & Heisey, 2001; O’Keefe, 2007). We created two artificial LMMs using the *simr* package in R (Green & Macleod, 2016), one corresponding to the free‐ranging population and another corresponding to the mesocosm experiment. In the former, we replicated the terms and parameters of the standardized model exploring morph differences in home range size. In the latter, we replicated the terms and parameters of the standardized model exploring morph differences in social dominance (see Appendix S1). Following Green and Macleod (2016), we then modified the standardized estimate for the morph factor (i.e., effect size) to either 0.5 or 0.8, and conducted a power analysis by running 1,000 simulations at 10 different levels of sample size (range = 5–50 lizards within each morph).

## 3 RESULTS

### 3.1 Spatial behavior in a free‐ranging population of *Podarcis muralis*

In total, we accumulated 5,046 sightings of 472 different lizards. Eighty‐seven lizards were observed more than 1 year (maximum = 3 years, 21 lizards). Out of those, 76 (87.4%) were found on the same wall as the previous year, seven (8%) moved between neighboring walls, and only four (4.6%) changed to a nonadjoining wall between years. Only 181 males and 101 females were large enough (SVL ≥ 56 mm) to be included in the analyses about morph differences (Table S2). For each variable considered, we provide separate measures of centrality and dispersion for males and females in Table S3. Residents represented 59.6% of both adult male and female lizards, and no color morph was overrepresented among resident or transient lizards (GLMM (binomial): *χ^2^* = 1.60, *p = *.81). Movements between walls were similarly frequent among color morphs (GLMM (gamma), *χ^2^* = 2.80, *p* = .59). Color morphs did not differ in the total number of resightings accumulated, the mean days elapsed between consecutive resightings, or the mean distance between consecutive relocations (*p* > .28; see Table S4 for more details and effect size).

We could calculate reliable estimates of linear home‐ and core ranges for 83 lizards, but decided to exclude mixed‐morph lizards from the analyses due to their scarcity. The final dataset consisted of 70 lizards: 18 females and 52 males with at least 17 resightings (Table S2). Neither sex showed significant differences in SVL among color morphs (LMM: *χ^2^* = 6.61, *p* = .16). Males had both larger linear home ranges and core ranges than females, and also perched higher on the stone walls (*p* < .01; Table S4). Morphs did not differ in the size of their home‐ and core ranges, neither in males (LMM: home ranges: *χ^2^* = 4.31, *p* = .19; core ranges: *χ^2^* = 2.41, *p* = .30), nor in females (LMM: home ranges: *χ^2^* = 0.44, *p* = .80; core ranges: *χ^2^* = 3.09, *p* = .21). Similarly, mean perching height did not differ among color morphs (*χ^2^* = 1.01, *p* = .60; Table S4). In males, we did not find significant intermorph differences in the number of females within their linear home‐ or core range (GLMM (gamma): *χ^2^* < 1, *p* > .3). Likewise, males of different color morphs did not differ in the size of their exclusive ranges (i.e., the fraction of core range that is not shared with any other male) or in the number of female core ranges partially included within those ranges (*p > *.35; Table S4).

### 3.2 Mesocosm experiment

#### 3.2.1 Morphology and color traits

None of the morphometric traits examined (reported to be under intrasexual selection in male wall lizards; Baird, 2013; Pérez i de Lanuza, Carazo, & Font, 2014; While et al., 2015) were found to differ among color morphs in our sample of experimental males (Table S5). In females, neither SVL nor ILL (both positively correlated with fecundity; Kratochvíl, Fokt, Rehák, & Frynta, 2003; Olsson et al., 2002) varied with color morph, but white morph females (before reproduction) were found to be significantly heavier than orange females (Table S5).

#### 3.2.2 Spatial behavior

**Figure 3:**
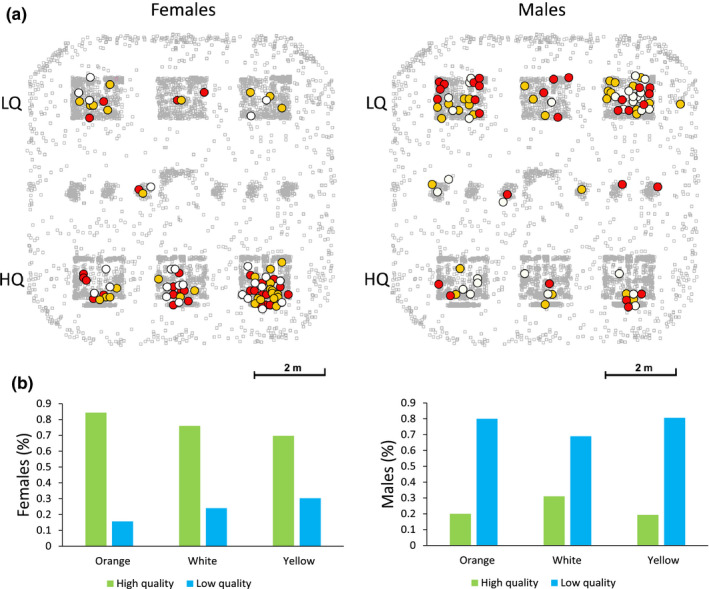
Distribution of the lizards among high‐ and low‐quality sites in the experimental enclosures. (a) Position of the peak density of resightings for each male and female (filled circles), plotted on a background schematic diagram of an experimental enclosure obtained by pooling together every resighting of a lizard collected during the experiment (gray squares). The orange, white, or yellow fill of the circles represents color morph. (b) Barplots showing the relative frequency of males and females of each color morph that settled in high‐ or low‐quality sites

Overall, we accumulated 7,190 resightings of the marked lizards in 655 scan samplings. The total number of resightings per lizard differed significantly between sexes (males were resighted more often), but not among color morphs (GLMM (negative binomial): sex: *χ* ^2^ = 57.11, *p < *.001; morph: *χ* ^2^ = 0.81, *p = *.67). Likewise, we found a strong intersexual difference in the ability to settle in high‐ or low‐quality sites, but no intermorph difference (GLMM (binomial): sex: *χ* ^2^ = 56.38, *p < *.001; morph: *χ* ^2^ = 1.37, *p = *.50; Figure 3). In fact, even though lizards were evenly distributed among sites (HQ: *N = *91, LQ: *N* = 89), females had three times higher odds of settling in HQ sites (OR = 3.26), whereas only highly dominant males managed to occupy HQ sites (Figure S4). Specifically, an increase of one *SD* in social dominance among males meant 4.5 times higher odds of settling in HQ sites (*p* < .001; Table S6). Males settled in HQ pallets did not differ in body size, weight, or head variables from males settled in LQ pallets (LMM: *χ* ^2^ < 1, *p > *.2).

As expected, males had larger home‐ and core ranges than females, and lizards settling in HQ sites occupied smaller areas than lizards in LQ sites (LMM on k50: sex *χ* ^2^ = 34.95, *p < *.001; pallet quality: *χ* ^2^ = 7.64, *p = *.006). In males, variation in home‐ and core range size was significantly explained by social dominance (*p* < .001; Table S6), but not by color morph (*p* > .20; Table S6). In females, we found significant differences in home‐ and core range areas among female color morphs, with white morph females showing the largest areas (*p < *.001; Table S7). Male–female spatial overlap was not affected by color morph, but was significantly associated with site quality in both sexes (*p < *.01; Tables S6 and S7). Males established in HQ sites overlapped with 3.0 ± 1.2 more females, and females established in LQ sites overlapped with 1.7 ± 1.0 more males.

#### 3.2.3 Intrasexual competition

**Figure 4:**
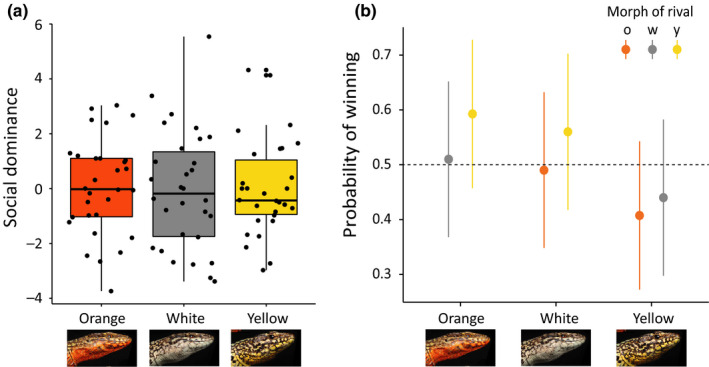
Male–male competitive interactions. (a) Boxplot of social dominance by color morph. Boxes indicate the interquartile range (IQR, 50% of data). Horizontal lines represent the median, and bars extend to 1.5 times the IQR. A jittered dot cloud shows the value of the variable of interest for each lizard in our dataset. (b) Mean plot showing the probability of winning for each morph combination according to the predicted values of the logistic mixed models. Bars extend to the CI_95%_. The horizontal dotted line marks 50% probability

We recorded 927 intrasexual interactions (614 in males and 384 in females). Competitive interactions were more common among males (*N = *543; 88% of total male–male interactions) than among females (*N* = 25; 7%), which were often observed in groups engaged in prolonged coperching in the vicinity of a male (*N* = 338, 88%). In males, display posturing and/or foot shakes (IIa, appeasement, Table 1) were observed in 60% of these competitive encounters, a third of them (36%) ended with a rapid chase/flight, and 16% involved physical aggression (i.e., bites). Display behavior and bites were usually exhibited only by the winning lizard (display: *N = *307, 91% only by winner; bite: *N* = 89, 70% only by winner), while foot shakes were almost exclusively performed by losing males (*N* = 70, 93% only by loser) with no differences among morphs (*χ* ^2^ = 3.07, *p = *.22). No morph combination was overrepresented among these contests (*χ* ^2^ = 5.63, *p* = .40). We found no evidence of an intermorph difference in the index of social dominance estimated from the Bradley–Terry model (*p* = .68; Table S6 and Figure 4). After dealing with pseudoreplication (200 different pairs of rivals; Table S8), we found no effect of morph combination on the outcome of heteromorphic contests (GLMM (binomial): orange: *χ* ^2^ = 0.33, *p* = .56; white: *χ* ^2^ = 1.83, *p* = .18; yellow: *χ* ^2^ = 0.88, *p* = .35). In fact, for either of the morphs involved in these combinations, the probability of winning did not differ significantly from even odds (Figure 4).

#### 3.2.4 Male–female interactions and parentage

In total, we recorded 1,230 male–female interactions, of which 1,098 were deemed as reproductive because they involved the exchange of sex‐specific behaviors (441), prolonged coperching (551), and/or copulatory behavior (153).

**Figure 5:**
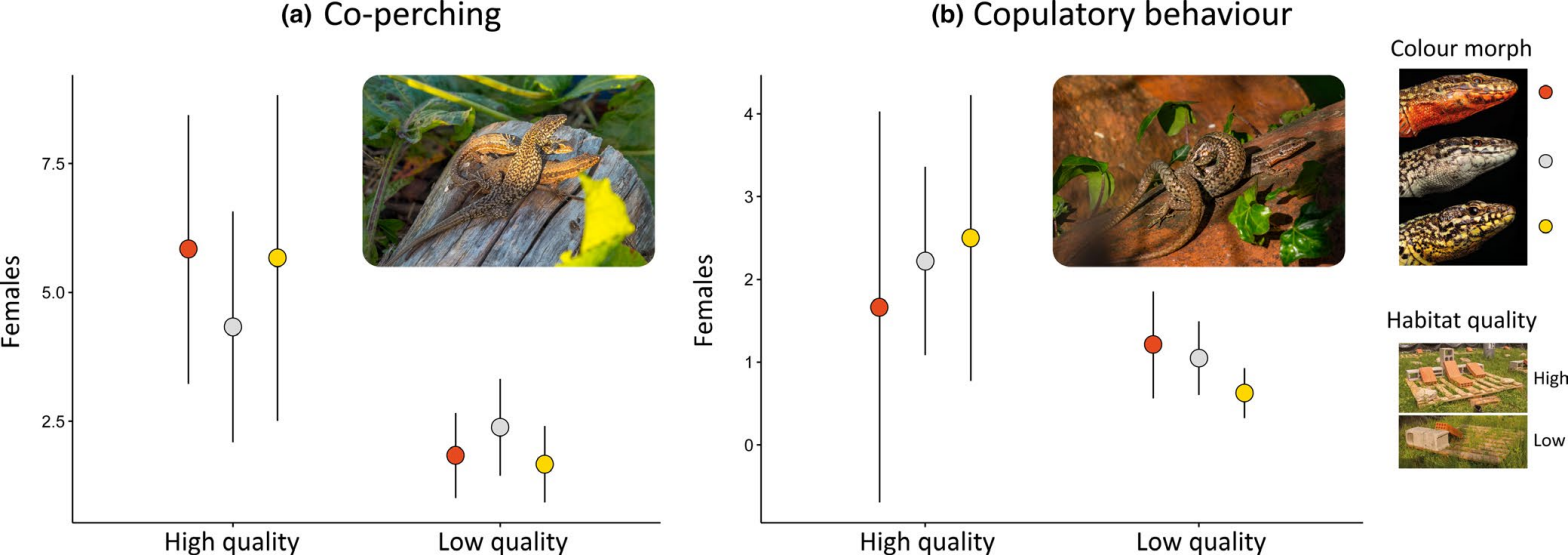
Variation in the number of different females with which males engaged in either coperching (a) or copulatory behavior (b, copulation and tail grabs). Males settled in high‐quality pallets interacted with a significantly higher number of individual females, while male color morphs did not differ in sociosexual behavior. Bars extend to the CI_95%_

**Figure 6:**
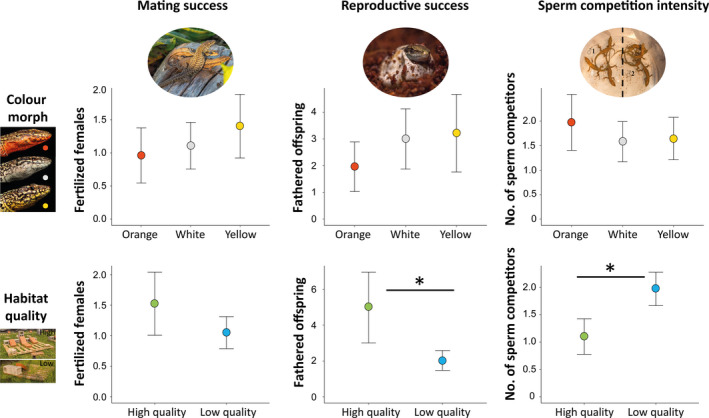
Variation in male individual fitness among alternative color morphs (up) and between sites of different quality (below). Bars extend to the CI_95%_. Significant differences are marked with an asterisk (*p < *.001)

Male color morphs did not differ in the number of females with which they interacted, engaged in coperching, or engaged in copulatory behavior (*p > *.57; Table S9). Unsurprisingly, males settled in HQ sites engaged in reproductive interactions more frequently (LMM: *χ^2^* = 36.91, *p < *.001) and with a higher number of females than males settled in LQ sites (*p < *.001; Table S9; Figure 5). We found no difference in relative reproductive success or relative mating success among male color morphs (*p > *.19; Table S9). Males settled in HQ sites showed significantly higher relative reproductive success (*p* < .001), but not relative mating success (*p* = .107; Table S9). Sperm competition intensity faced by each individual male was also independent of color morph (*p = *.56), but significantly higher in low‐quality sites (*p = *.001; Table S9). No morph combination in male cosirings was more prevalent than expected by chance (*χ* ^2^ = 2.13, *p = *.83; Table S10). Results from the analysis of male fitness are summarized in Figure 6.

Female color morphs did not vary in the number of males encountered in reproductive interactions, eggs produced, or fertilization success (*p > *.11; Table S11). Body mass and ILL (but not color morph, *p = *.71) were significantly related to laying date, with heavier and longer females laying their clutches sooner than the rest (*p = *.014; Table S11). Although we found high levels of multiple paternity within the experimental enclosures (81% of clutches), female color morphs did not differ in the number of sires fathering offspring in their clutches (LMM: *χ* ^2^ = 2.84, *p = *.24), nor in the number of viable juveniles conceived (LMM: *χ* ^2^ = 4.31, *p = *.12). Relative measures of fitness yielded similar results (*p > *.16; Table S11). We found a significant effect of habitat quality on some aspects of female social behavior and reproductive parameters: Females established in LQ sites interacted with a higher number of males showed higher levels of multiple paternity, and their clutches contained a smaller fraction of unfertilized eggs (*p* < .05; Table S11).

**Figure 7:**
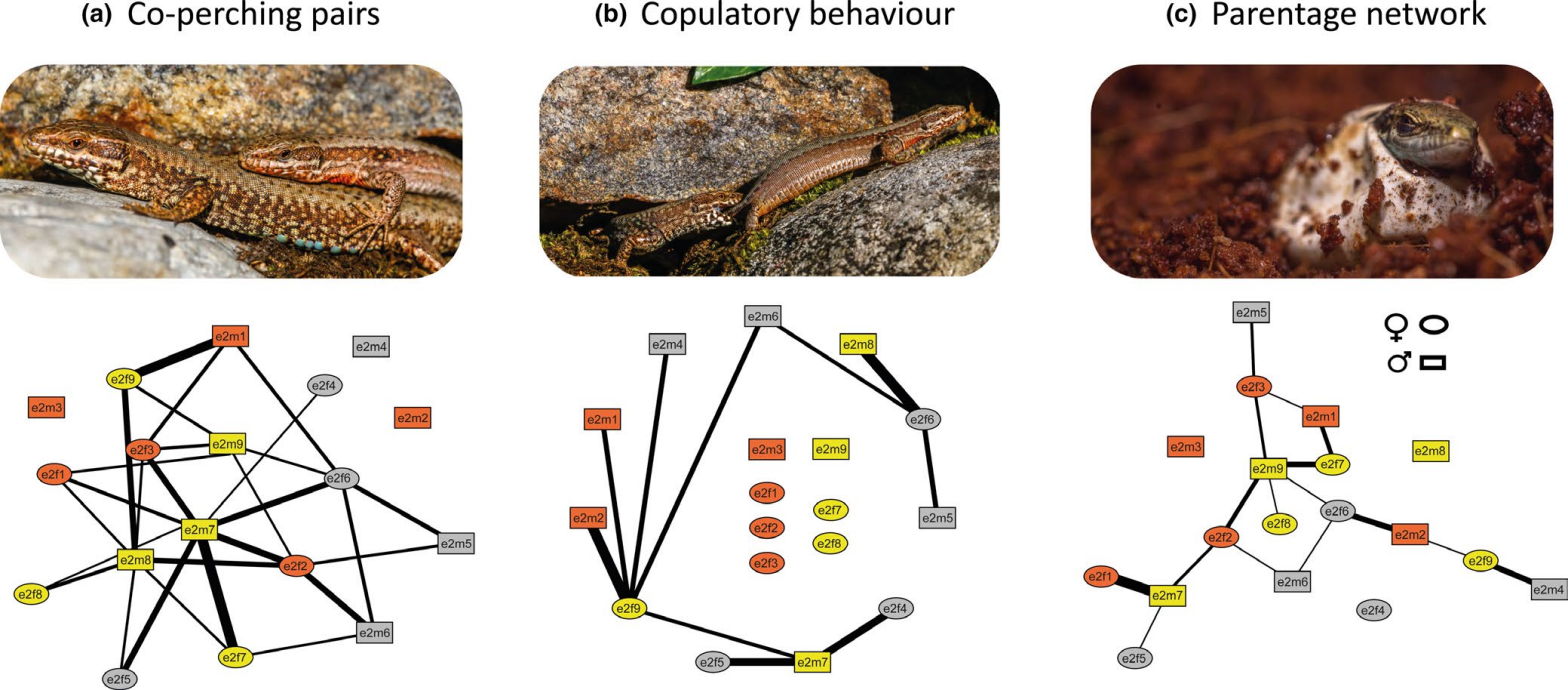
Example network diagrams from one of our experimental enclosures based on (a) coperching pairs, (b) copulatory behavior (i.e., interactions involving tail grabs and/or matings), and (c) the resulting parentage network. Each node represents an individual lizard, with shape and color denoting sex and color morph, respectively. Alphanumeric codes within the nodes correspond to the unique ID of each lizard within the enclosure. The thickness of the lines connecting nodes characterizes the number of social interactions (a, b) or offspring (c) between each dyad of lizards. Unconnected nodes represent lizards that we did not observe to engage in coperching or copulatory behaviors (a, b), or did not reproduce (c)

Parentage was significantly predicted across enclosures by both of the association networks based on social behavior during male–female interactions (coperchings: *χ* ^2^ = 51.91, *p* < .001; copulation attempts: *χ* ^2^ = 45.40, *p* < .001). However, neither of the behavioral association networks nor the resulting parentage network were found to be affected by morph combination (coperchings: *χ* ^2^ = 0.69, *p* = .69; copulation attempts: *χ* ^2^ = 0.83, *p* = .83; parentage: *χ* ^2^ = 0.32, *p* = .32; Figure 7). We found a significant interaction of the parental morph combination over juvenile body mass (LMM: *χ* ^2^ = 12.91, *p = *.012). Splitting the dataset by female morph, we found that this result was exclusively driven by a nonsignificant tendency of yellow males to sire heavier offspring than orange males when coupled with white females (LMM: *χ* ^2^ = 6.28, *p = *.09). We found no effect of male or female morph alone on juvenile mass (LMM: *χ* ^2^ < 1, *p* > .5).

#### 3.2.5 Power analysis

The sensitivity analysis in G*Power estimated a minimum detectable effect size of Cohen's *d* = 0.46 (*N = *181) and Cohen's *d* = 0.88 (*N* = 52) for activity and space use differences (respectively) between male color morphs in the free‐ranging population. For the mesocosm experiment, we estimated that a sample size of 90 males and females would allow us to detect medium‐sized (Cohen's *d* = 0.66) intrasexual differences in behavior and fitness among color morphs with a standard statistical power of 0.80. These effect sizes are at the lower end of the range of effect sizes (Cohen's *d* = 0.49–2.32), which we calculated from the literature (Table S1), suggesting that we had enough statistical power to detect even subtle but biologically meaningful differences among morphs. Accordingly, results from the two simulation‐based analyses of power showed that our sample sizes were high enough to detect biologically relevant differences among color morphs (power > 0.80 to detect medium‐sized and large effect sizes). In fact, introducing the observed coefficients for the fixed and random factors in the simulations and plotting the expected increment in power at different sample sizes revealed a higher statistical power for the data presented here than the more conservative estimates obtained in G*Power (Figure S5).

## 4 DISCUSSION

Overall, our results from both a longitudinal field study and an enclosure experiment argue against the hypothesis that *P. muralis* color morphs reflect alternative reproductive strategies (ARS) involving differential sociosexual behavior and space use. In territorial species such as *Podarcis* lizards, resource‐holding potential, spatial behavior, and activity are expected to vary across males employing alternative strategies (Calsbeek & Sinervo, 2002a, 2002b; Molnár, Bajer, Szövényi, Török, & Herczeg, 2016; Noble, Wechmann, Keogh, & Whiting, 2013; Sinervo et al., 2000; Sinervo & Svensson, 2002; Sinervo & Zamudio, 2001; Zamudio & Sinervo, 2000). In this study, we did not find any evidence that color morphs differ in resource‐holding potential (i.e., social dominance, agonistic behavior, territoriality), space use (i.e., site fidelity, home range size, overlap with conspecifics), or activity (i.e., frequency of resightings, distance between consecutive resightings).

No color morph was overrepresented among resident or transient lizards in the field, and we did not observe differences in either intermorph resighting propensity, distance between consecutive resightings, or interannual site fidelity. Furthermore, color morphs showed similar home range size and male–female overlap both in natural conditions and in experimental enclosures. In both sexes, alternative color morphs obtained similar relative fitness within the enclosures (which would be necessary for their maintenance over time), but crucially, this was not associated with different behavioral strategies. In line with previous evidence on the behavioral ecology of territorial lizards (Baird, 2013; Baird, Timanus, & Sloan, 2003), males competed fiercely to settle in high‐quality sites irrespective of their color morph, and the subset of successful dominant males (23%) engaged in coperching with a higher number of females, experienced significantly lower levels of sperm competition, and ultimately achieved higher reproductive success. In sum, while lizards were strongly attracted to high‐quality sites (both in the field and in the mesocosm experiment), we did not find any evidence that color morph played a role in securing access to them or in the ability to exclude other conspecifics from its use. In fact, we did not find an effect of color morph on the outcome of male–male competitive interactions. These results contrast previous evidence suggesting lower fighting ability in orange morph males during laboratory‐staged encounters (Abalos et al., 2016), likely because any differences between size‐matched morphs meeting at a neutral arena are overridden by the effect of size asymmetries and residency status when confrontations occur under more natural conditions (Stuart‐Fox & Johnston, 2005). Similarly, Sacchi et al. (2009) reported no effect of color morph on aggressive behavior during laboratory‐staged contests when the experimental design allowed for size and residency asymmetries. Previous studies have reported larger body sizes in orange morph lizards with respect to white (Calsbeek et al., 2010; Sacchi et al., 2007), with some authors suggesting an advantage of orange morph lizards in male–male competition for preferred territories and hence reproductive success (Calsbeek et al., 2010). The size difference, however, may result from miscategorizing subadult lizards as pertaining to the white morph (i.e., the lizards' ventral surface appears white to the human eye before achieving sexual maturity), leading to the conflation of any possible morph difference with the expected size asymmetry between younger and older lizards. For instance, orange morph lizards from our study population in Angoustrine are only 1.7 ± 0.3 mm larger than white morph lizards in the free‐ranging population of Angoustrine (1942 adult SVL > 56 mm lizards), which represents a 2.6% of the average SVL in adult lizards. There is, in fact, no evidence for biologically relevant differences among male *P. muralis* morphs neither in morphology or sex‐specific coloration (i.e., UV‐blue ventrolateral spots; Pérez i de Lanuza et al., 2014), and in this study, we did not observe differential use of agonistic behaviors during intrasexual competitive interactions.

The existence of ARS in a polymorphic territorial species does not necessarily imply that color morphs must differ in territoriality or aggressive behavior (Shuster & Wade, 2003). ARS in males of polygynandrous species are often expressed as differential sexual behaviors (e.g., mate‐guarding) or physiological adaptations (e.g., increased testis size) representing alternative solutions to the trade‐off between securing fertilizations and acquiring new mates (Formica et al., 2004; Shuster, 2008; Taborsky, 2001; Taborsky & Brockmann, 2010). For example, in the Australian painted dragon (*Ctenophorus pictus*), yellow morph males have larger testis and strongly outperform orange males in laboratory‐staged sperm competition trials, despite the absence of differential territory‐acquisition abilities between both morphs (Healey & Olsson, 2008; Olsson, Schwartz, Uller, & Healey, 2009). In contrast, *P. muralis* male morphs within experimental enclosures showed similar time allocation between guarding females and acquiring new mates, and no difference in the number of mates sired, and experienced similar levels of sperm competition. In *U. stansburiana*, the interplay between the usurper, guarding, and sneaker strategies leads to morph‐biased patterns of shared paternity, with yellow sneaker males obtaining almost all of their reproductive success from cosiring clutches with orange males, while blue guarding males show low overall levels of cosiring (especially with yellow males; Sinervo & Zamudio, 2001; Zamudio & Sinervo, 2000). Here, we found no evidence of a similar bias, with no morph combination in cosired clutches being more prevalent than expected by random association. In fact, given the absence of differences in precopulatory behavior, the similar reproductive success achieved by males of the three color morphs indirectly argues against the existence of physiological adaptations in the context of postcopulatory sexual selection (e.g., larger testis and ejaculates, which would have biased paternity in the absence of differential social behavior). Further research could directly address this question by studying reproductive physiology in *P. muralis* color morphs and staging realistic sperm competition trials across morphs.

While most research on color polymorphism and ARS concerns males, females are also often polymorphic. Differential female breeding strategies, such as the different solutions to the trade‐off between egg size and number described in the female color morphs of *U. stansburiana* (Alonzo & Sinervo, 2001), have also been suggested to occur in *P. muralis*. One study of an Italian population found that, in captivity, yellow females laid relatively larger clutches of smaller eggs than white morph females (Galeotti et al., 2013). Our results also contradict this hypothesis, as we found no difference among female morphs in clutch size or juvenile mass. Unexpectedly, white morph females roamed across larger areas than females from the other morphs. Rather than alternative strategies in space use, we think this difference may result from white morph females being heavier (and likely more advanced in their ovarian cycle) when released into the enclosures. This could have prompted exploratory behavior in the search for suitable egg‐laying sites earlier in this morph. Whether this unexpected result is artefactual or derives from differences in the timing of reproduction among female morphs should be examined in future studies. Overall, our results constitute strong evidence against the existence of ARS concerning male–male aggression, spatial dominance, sexual behavior, or breeding strategy in *P. muralis* color morphs.

Even if color morphs do not reflect ARS, nonrandom mating with respect to color can contribute to the stability of polymorphic systems over time (Galeotti et al., 2003; Roulin, 2004; Wellenreuther et al., 2014). Mate preferences may vary among individuals if the expected benefits derived from mating with differently colored individuals are a function of the chooser's morph (e.g., genetic compatibility) or vary relative to other factors (e.g., time, space, population density; Mckinnon & Pierotti, 2010; Roulin, 2004; Wellenreuther et al., 2014). In polymorphic Pyrenean populations of *P. muralis*, homomorphic pairs of males and females occur more frequently than heteromorphic pairs, irrespective of local morph diversity (Pérez i de Lanuza et al., 2013; Pérez i de Lanuza, Font, & Carretero, 2016). This assortative pairing suggests a role of color morph in mate choice, but is not sufficient to demonstrate its existence (Roulin & Bize, 2007; Roulin, 2004; Wellenreuther et al., 2014). In fact, color‐assortative pairing can also occur in the absence of mate choice, for example, if phenotypically similar lizards tend to cluster together within populations as a consequence of similar environmental constraints or population viscosity (Roulin, 2004; Wellenreuther et al., 2014). Here, we did not find evidence of morph assortativity in the male–female social interactions observed within the enclosures. Previous research using laboratory‐staged mate choice trials has already reported the absence of color‐assortative preferences toward differently colored males in *P. muralis* females (Sacchi et al., 2015). However, we think that our results constitute a more realistic perspective of male–female dynamics in nature, since mounting evidence suggests that the initiation and outcome of precopulatory male–female interactions in lizards are almost completely under male control (Andrews, 1985; Heathcote et al., 2016; Noble & Bradley, 1933; Olsson, 2001; Olsson & Madsen, 1995; Olsson et al., 2013; Tokarz, 1995). Following our results, we deem unlikely that the color‐assortative pattern observed in the wild (>60% of pairings at our study site; see Pérez i de Lanuza et al., 2013) results from the lizards actively choosing to pair with similarly colored partners. Rather, assortative pairing could result indirectly from some form of clustering in the spatial distribution of color morphs in natural populations, due to population viscosity or ecophysiological constraints (Lindsay et al., 2019; Pérez i de Lanuza, Sillero, et al., 2018; Svensson, 2017; Svensson, Abbott, Gosden, & Coreau, 2009; Wellenreuther et al., 2014).

Our results also offer evidence against the existence of strong frequency‐dependent effects on morph fitness. As stated before, by introducing the color morphs in equal frequencies within the enclosures we simulated a situation that is rarely observed in any of the different *P. muralis* lineages showing color polymorphism. Such balanced morph frequencies were never observed in natural populations from eastern Pyrenees (examined in Pérez i de Lanuza et al., 2017, Pérez i de Lanuza, Sillero, et al., 2018, *N* = 116 localities), where white morph lizards usually predomínate (e.g., morph frequency ranges: orange = 0%–60%; white = 27%–92%; yellow = 0%–25%; orange‐white = 0%–27%; yellow‐orange = 0%–13%), and only 3.45% of the localities show a morph other than white as the most common. Additionally, morph frequencies do not seem to experience substantial interannual variation, with the same rank order being maintained in the study population of Angoustrine for the last 6 years (Figure S1). If color morphs are, in fact, under some form of frequency‐dependent selection, the frequencies observed in natural populations may reflect a selective equilibrium where each morph obtains equal average fitness. By using a 1:1:1 morph ratio in our experimental setup, we simulated a displacement from such equilibrium frequencies, which should have resulted in a selective pullback, and hence higher fitness in white morph lizards (Roulin, 2004; San‐Jose et al., 2014; Sinervo et al., 2007; Svensson, 2017). In contrast, we did not find significant differences in fitness among color morphs, suggesting that strong frequency‐dependent effects on morph fitness are unlikely to be the prime determinant of morph relative frequencies in *P. muralis* natural populations. This study is primarily aimed at detecting differences in sociosexual behavior among male morphs, and we acknowledge that our experimental design is not tailored to test for frequency‐dependent effects on fitness. In fact, testing for a rare (NFDS) or a common morph advantage with a mesocosm design would require to introduce each morph consistently in lower or higher frequency across the enclosures (Roulin, 2004; Svensson, 2017; Wellenreuther et al., 2014). Additionally, selection on color morphs is often dependent on both biotic (demography, sex ratio) and abiotic factors (environmental conditions), as well as on the population morph composition and relative morph frequencies (Forsman, Ahnesjö, Caesar, & Karlsson, 2008; Gosden & Svensson, 2008, 2009; McLean & Stuart‐Fox, 2014; McLean, Stuart‐fox, & Moussalli, 2015; Svensson, 2017; Svensson et al., 2020; Willink et al., 2019). Future studies should examine the environmental dependence of morph fitness in populations characterized by extreme morph compositions and socioecological contexts (i.e., varying sex ratio, density, and environmental conditions), for example, by combining field observations with the experimental alteration of these same parameters in enclosure experiments.

The maintenance of color polymorphism may be possible through genetic mechanisms entirely independent of sociosexual behavior. For instance, if heterozygosity at genes coding for color polymorphism provides fitness benefits (i.e., overdominance), and the advantages of heterozygosity only concern viability selection (e.g., survival to adulthood), color morphs would be maintained in the population even if morphs mated at random (Krüger, Lindström, & Amos, 2001; Roulin & Bize, 2007; Roulin, 2004; Wellenreuther et al., 2014). In a breeding experiment conducted on captive *P. muralis* lizards from Italian polymorphic populations, morph pair combination was found to affect fertilization success, hatching success and newborn quality (i.e., juvenile mass; Galeotti et al., 2013). Here, we found a weak effect of color morph combination on juvenile mass, but the low sample size (*N = *44) is insufficient to draw firm conclusions. To examine the role of genetic compatibility and overdominance on stabilizing color polymorphism in future research, we would need to estimate juvenile fitness and interannual survival at the genotypic (rather than the phenotypic) level, as the fitter heterozygotes could be phenotypically indistinguishable from other genotypes (Gratten et al., 2008; Johnston et al., 2013; Tregenza & Wedell, 2000).

Despite drawing substantial interest from evolutionary biologists, the evolutionary causes and consequences of lacertid color polymorphisms are still poorly understood. Alternative reproductive strategies have been suggested to occur in the Dalmatian wall lizard (*Podarcis melisellensis*), where orange males have been found to present larger body size, disproportionately large heads, and higher fighting ability in size‐matched contests staged in the laboratory (Huyghe et al., 2007, 2009; Huyghe, Vanhooydonck, Herrel, Tadić, & Van Damme, 2012). In contrast, in the European common lizard (*Zootoca vivipara*), interpopulation differences in morph composition and rapid morph cycles have been explained by the cumulative effect of two frequency‐dependent mechanisms starkly different from ARS (morph‐biased female mate choice and offspring survival; San‐Jose et al., 2014; Sinervo et al., 2007). Meanwhile, differences in morph composition among island populations of the Skyros wall lizard (*Podarcis gaigeae*) have been found to be fall within that expected under neutral genetic divergence, and genetic drift could thus not be rejected as an explanation of the pattern (Runemark et al., 2010). Lastly, most of the evidence suggesting the existence of physiological or behavioral morph differences in *P. muralis* comes from studies conducted on the southern Alps sublineage (Galeotti, 2013; Sacchi, Mangiacotti, et al., 2017; Sacchi, Scali, et al., 2017; Galeotti et al., 2007; Scali et al., 2016), which is only distantly related to the western European lineage found in Pyrenees (Gassert et al., 2013; Giovannotti, Nisi‐Cerioni, & Caputo, 2010; Schulte, Gassert, Geniez, Veith, & Hochkirch, 2012). These observations, together with the high prevalence and ancient origin of color polymorphisms in wall lizards (Andrade et al., 2019; Arnold, Arribas, & Carranza, 2007; Jamie & Meier, 2020), suggest the intriguing possibility that genes coding for the expression of the alternative color morphs might become linked to genes that influence other functionally relevant traits (i.e., physiology, behavior, life history, development) only at times, and hence be under selection only in some environments or in some lineages (i.e., *Podarcis* species). Linkage disequilibria are expected to decay rapidly if not counteracted by strong and chronic correlational selection, and genetic drift is very effective in leading to the loss of polymorphism (especially in small populations; Gray & McKinnon, 2007; Mckinnon & Pierotti, 2010; Sinervo & Svensson, 2002; Svensson, 2017). Hence, this evolutionary scenario would cause correlations between color and other phenotypic traits to vary either in space or in time, and even lead to morph loss in some populations or lineages. Polymorphism loss has likely occurred in wall lizards. Despite their putative ancestral origin (Andrade et al., 2019), color morphs are apparently absent in some *Podarcis* species (Speybroeck et al., 2016), and the polymorphic species that have been examined often show marked geographical variation in morph diversity (Jamie & Meier, 2020; MacGregor, Lewandowsky, et al., 2017; Pérez i de Lanuza, Sillero, et al., 2018; Runemark et al., 2010). However, due to its high genetic diversity, effective population sizes in *P. muralis* (and likely in other wall lizards) have been estimated to be sufficiently large (Ne > 4 × 10^6^; Yang et al., 2020) to allow for the long‐term persistence of a largely neutral trait under intermittent selection contingent on the environment. Local morph extinctions could thus be counteracted by immigration from larger populations where selectively neutral color expression could resist the eroding effect of genetic drift for longer periods, and interpopulation differences in morph composition would be mainly driven by the environmental and genetic constraints of color expression (Gray & McKinnon, 2007; Mckinnon & Pierotti, 2010; Roulin et al., 2004). Recent results showing the recessive genetic basis of orange and yellow ventral coloration in *P. muralis* with respect to white (Andrade et al., 2019) could provide a simple explanation for the marked bias toward the white morph observed in natural populations (Pérez i de Lanuza et al., 2017, Pérez i de Lanuza, Ábalos, et al., 2018, Pérez i de Lanuza et al., 2019; Figure S1). Future research should investigate the possibility of spatially or temporally varying correlations between polymorphic color expression and other phenotypic differences in *Podarcis* lizards, as well as evaluate the relative importance of selection and genetic drift in shaping interpopulation differences in morph composition and relative frequencies (Runemark et al., 2010).

In conclusion, our results do not warrant the frequent assumption that behavioral ARS underlie the maintenance of ventral color morphs in the European common wall lizard. In the wake of the *U. stansburiana* model, much effort has been devoted to detect intermorph differences suggestive of behavioral ARS in polymorphic lizards (Calsbeek et al., 2010; Fernández et al., 2018; Healey, Uller, & Olsson, 2007; Yewers et al., 2016). However, these studies have often painted a much more complex picture involving several evolutionary processes, of which ARS may represent but one in many mechanisms explaining the vast diversity of lizard color polymorphisms (Carpenter, 1995; Huyghe et al., 2012; McLean et al., 2015; San‐Jose et al., 2014). We should therefore reassess the allegedly central role of ARS in explaining the maintenance of phenotypic variability in nature, and broaden the perspective to incorporate other hitherto overlooked processes.

## CONFLICT OF INTEREST

None declared.

## AUTHOR CONTRIBUTIONS

**Javier Abalos:** Conceptualization (equal); data curation (lead); formal analysis (lead); investigation (lead); methodology (lead); visualization (lead); writing–original draft (lead); writing–review and editing (equal). **Guillem Pérez i de Lanuza:** Conceptualization (equal); supervision (equal); validation (equal); writing–review and editing (equal). **Alicia Bartolomé:** Data curation (equal); investigation (equal); methodology (equal); validation (equal); writing–review and editing (equal). **Océane Liehrmann:** Investigation (equal); methodology (equal); writing–review and editing (supporting). **Hanna Laakkonen:** Investigation (equal); methodology (equal); writing–review and editing (supporting). **Fabien Aubret:** Conceptualization (equal); validation (equal); writing–review and editing (equal). **Tobias Uller:** Conceptualization (equal); validation (equal); writing–review and editing (equal). **Pau Carazo:** Conceptualization (equal); validation (equal); writing–review and editing (equal). **Enrique Font:** Conceptualization (equal); supervision (equal); validation (equal); writing–review and editing (equal).

## Supporting information

Figure S1

Figure S2

Figure S3

Figure S4

Figure S5

Video S1

Appendix S1

## Data Availability

The datasets used and analyzed in this study are available from Dryad: https://doi.org/10.5061/dryad.j0zpc86bx
