# Supplementary figures and images for: No evidence for differential sociosexual behavior and space use in the color morphs of the European common wall lizard (Podarcis muralis)
Source: Ecol Evol. 2020 Oct 8;10(20):10986–1005. doi: 10.1002/ece3.6659 (PMC7593164; doi:10.1002/ece3.6659)

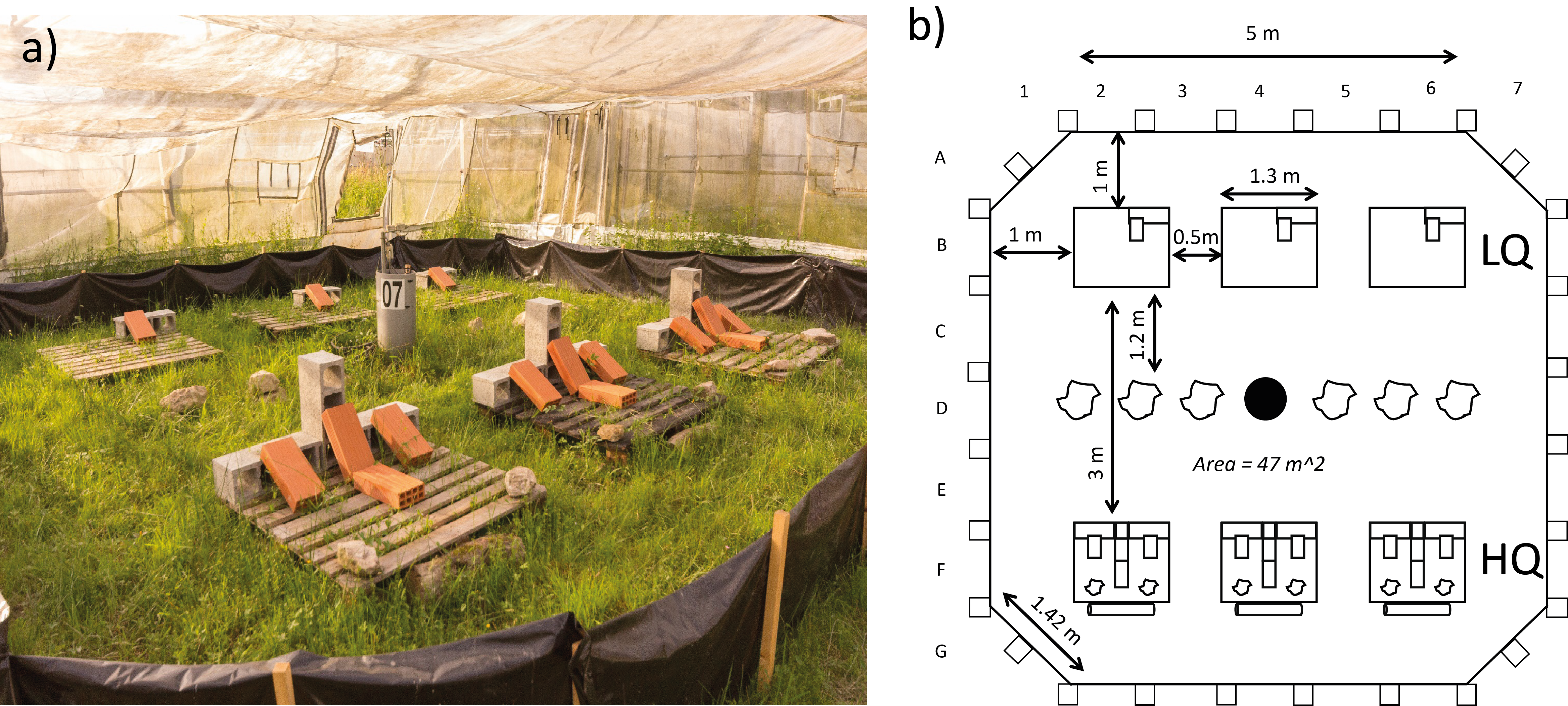

Supplement: Supplementary file 3 — Figure S3 [file ECE3-10-10986-s003.png]

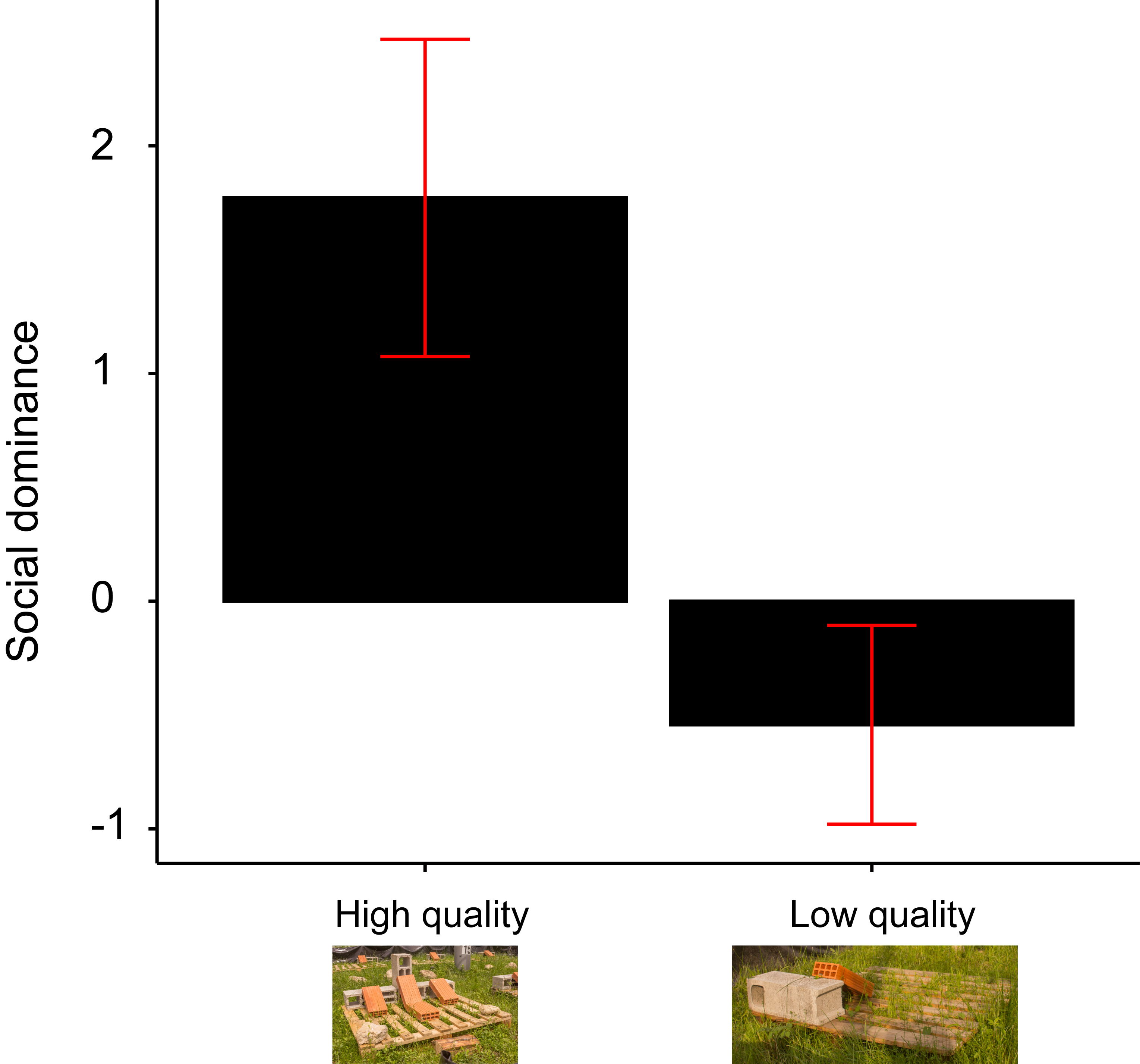

Supplement: Supplementary file 4 — Figure S4 [file ECE3-10-10986-s004.png]
