# Supplementary material for: No evidence for differential sociosexual behavior and space use in the color morphs of the European common wall lizard (Podarcis muralis)
Source: Ecol Evol. 2020 Oct 8;10(20):10986–1005. doi: 10.1002/ece3.6659 (PMC7593164; doi:10.1002/ece3.6659)
Supplement: Supplementary file 7 — Appendix S1 [file ECE3-10-10986-s007.docx]

**Supplementary Material: Don’t judge a lizard by its colour: no evidence for differential socio-sexual behaviour and space use in the colour morphs of the European common wall lizard (*Podarcis muralis*).**

**Contents**

**Appendix S1:** Expanded materials and methods

**Supplementary Tables:** S1-S11

**Supplementary Figures:** S1-S5

**Supplementary Videos:** S1

**Appendix S1: Expanded materials and methods**

*Additional information on the study of spatial behaviour in a free-ranging population of P. muralis*

Lizards were identified by a combination of unique toe-clip markings, xylene-free permanent paint marks (Edding © 751, Edding Ltd, St Albans, UK), and/or by natural individual traits (see Pérez i de Lanuza *et al.,* 2014 for additional details). During the natural peak activity hours (10.00-13.30 and 15.30-19.00 local time) we carried out surveys (45 - 90 min, 650-1300 m) where we collected data on the spatial position of marked lizards (at least 2 h between consecutive rounds). We used short focus binoculars (Papilio 8.6 x 21, Pentax, Tokyo, Japan) and a laser rangefinder (Disto A6 and A8, Leica Geosystems, St. Gallen, Switzerland; m.e. = ± 1 mm) to record lizards’ position with respect to mapped landmarks. Then, using Autocad 2013 (Autodesk Inc., San Rafael, Ca, USA) we created a scaled map of the study site and a simplified diagram of the lateral face of each of the stone-walls (Fig. 1). This sampling methodology provided two types of data: frequency of re-sightings, used as a proxy of activity levels (DeNardo & Sinervo, 1994; Sinervo *et al.*, 2000; Noble *et al.*, 2013), and positional data, used to estimate space use (home- and core-range size) and overlap with conspecifics (Boag, 1973; Heathcote *et al.*, 2016).

*Additional information on female housing and hibernation before the experiment*

We installed an anti-bird net covering each of the 33 tanks used, which prevented avian predators while allowing local invertebrates to get inside the tanks and serve as primary food source. To increase habitat complexity, we complemented the naturally occurring vegetation inside the tanks by providing a perforated brick to serve as shelter, two wood logs, and a water dish to collect rain-water. We visited the tanks monthly to trim the weeds and provide an additional supply of food (*Tenebrio molitor* larvae and *Acheta domesticus* adults). On February 15 2018, we re-captured the females within the outdoor tanks, installed them in individual hibernation pods consisting of a plastic tub (12 cm diameter, 8 cm high) covered with a perforated lid and filled with moist coco husk, and stored them for 10 weeks in a dark climatic chamber to mimic the conditions at their capture sites (1^st^ week: 10ºC/ 2^nd^-9^th^ weeks: 5ºC/ 10^th^ week: 10ºC).

*Additional information on the housing of gravid females after the experiment*

We housed females individually in 55x38x28 cm high plastic terraria with a substrate of coco husk, a water dish, a shelter, and a small brick for basking over which we suspended a 40 W incandescent light (35ºC-18ºC temperature gradient). We also introduced a plastic tub filled with moist coco husk (11 cm of diameter, 8 cm deep) which the females used to lay the eggs. We set the light cycle to mimic field conditions during the reproductive season at the capture site (15L:9D), and additionally provided all lizards with full spectrum light (Reptistar 5.0: Sylvania, Danvers, MA, USA) for 2 h daily (12.00–14.00 h). We fed lizards two *Tenebrio molitor* larvae dusted with vitamins (JBL Terravit, Neuhofen, Germany) every other day. Overall, females stayed at the laboratory for less than 15 d before being released at their capture sites (i.e. after oviposition).

*Additional information on social network analysis*

To determine whether morph combination played a role in male-female social interactions, we ran Mantel permutation analyses on the compiled version of SOCPROG (Whitehead, 2009) on three different association networks based on i) male-female co-perching interactions, ii) copulatory behaviour (tail-grabs and mating, Table 1), and iii) parentage. We used social networks weighted by the total number of observed interactions (or the number of offspring, for parentage) between each dyad of lizards. We also tested for significant correlation between these two behavioural networks and the resulting parentage network in all of the ten experimental enclosures using Dietz R-tests (the non-parametric analogous of Mantel tests), recommended to correct for potential outlying values. All permutation analyses were based on 10000 permutations, which achieved stability in *P-*values. For each set of analyses (co-perching, tail-grab, and paternity networks), we combined the *P*-values from each different enclosure into a single test statistic using Fisher’s method (Fisher, 1932).

***Additional information on statistical analyses***

*Morphometry (mesocosm experiment)*

We tested for differences between the colour morphs in morphometry by fitting two linear mixed models (LMMs) with body size (SVL) and weight (mass) as response variables, colour morph as fixed factors, and capture locality as random factor. In males, we used similar models to explore the existence of inter-morph differences in head length (HL) and head width (HW). In females, we tested for differential inter-limb length (IIL) among colour morphs.

*Male-male competition (mesocosm experiment)*

We examined potential morph-differences in social dominance using a linear mixed models with colour morph, mass, head variables (HL, HW), and pallet quality (i.e. peak density of re-sightings at a high- or low-quality site) as fixed factors. Enclosure and capture locality were included as random factors. To explore nontransitive relationships of dominance, we created three separate logistic mixed-models (one for each colour morph) on the binary outcome of heteromorphic contests (1 = win, 0 = loss). In each of these models we included the opponent’s morph and identity as fixed and random factors, respectively. We also included the enclosure in which the interaction was observed as random factor. For the linear mixed-model we checked that all residuals conformed to homoscedasticity and normality assumptions. We used the glmer_dispersion function in the blmeco package of R to preclude potential problems of overdispersion in the residuals of the logistic GLMMs (ϕ ≈ 1 for every model).

*Spatial behaviour and activity (free-ranging population and mesocosm experiment)*

To determine the minimum sample size required to calculate a reliable estimate of home-range size, we subsampled the lizards with at least 25 sightings and graphed incremental range-observation plots showing the increase in linear home-range size as re-sightings are accumulated (Stone & Baird, 2002). For each lizard, we averaged the number of sightings when the curve reached 80% of final home-range size in four different incremental plots obtained by randomizing the order in which sightings were included (Rose, 1982; Stone & Baird, 2002). We then averaged the mean number of sightings for all the subsampled lizards, which resulted in a minimum sample size of 17 ± 1.84 sightings ( ± SEM) for reliable linear home-range estimation in the free-ranging population of Angosutrine, and 9 ± 1 sightings for the lizards in the enclosure experiment. Lizards with fewer sightings were deemed unsuitable to reliably estimate home-range size but were included in the analysis of spatial overlap.

1. *Free-ranging population*

We examined potential sex- and morph-differences in activity and spatial behaviour using mixed models with the number of re-sightings and the mean days elapsed between consecutive re-sightings, as well as the linear home-range, core-range, exclusive-range size, and mean perching height of each lizard as response variables. In each of these models we included SVL, colour morph, sex, and the interaction between the last two as fixed effects, and year as random factor. To test for the differences in spatial overlap, we fitted GLMMs with the number of either 1) males, or 2) females included within the home-, core-, or exclusive-range of each lizard as response variables, and the same predictors and random factors described for space use models. Whenever a significant sex difference was found, we run two additional models examining potential inter-morph variability separately. Home- and core-range size were logged to correct a weak positive skew, and analysed assuming a Gaussian distribution. Activity and spatial overlap variables showed a strong positive skew, which we corrected by fitting a gamma distribution with log-link function to the generalized linear mixed models. For each model, we checked that all residuals conformed to homoscedasticity and normality assumptions. For lizards observed for more than one year, we averaged the value of response variables.

1. *Mesocosm experiment*

For the analysis of activity and spatial behaviour, we first examined inter-sexual differences using mixed models with sex, morph, and the interaction between the latter as fixed factors. We then examined inter-morph differences by creating separate models for males and females. For males, we included colour morph, mass, head variables (HL, HW), and pallet quality (i.e. peak density of re-sightings at a high- or low-quality site) as fixed factors. In models on females, we included colour morph, mass, ILL, and pallet quality as fixed factors. Capture locality and enclosure were included as random factors in every model. We analysed activity using negative binomial GLMMs with the total number of re-sightings as response variable. Home- and core-range size were normally distributed and were analysed with LMMs. Competition for habitat quality was analysed by fitting a logistic GLMM on whether the lizard’s peak density of re-sightings was located at a high- (1) or low-quality (0) site. Male-female spatial overlap was analysed using GLMMs adjusted to a Poisson distribution, and the number of opposite-sex lizards showing core-core overlap as response variable. For each model, we checked that all residuals conformed to homoscedasticity and normality assumptions. We did not find over-dispersion problems in the Poisson or the negative binomial mixed-models (ϕ ≈ 1).

*Male-female interactions and individual fitness*

We used GLMMs fitted with a negative binomial distribution (or a Poisson distribution if the mean was equal to the variance) to explore potential inter-morph differences in the number of opposite-sex conspecifics with which the lizards were observed engaged in social interactions (i.e. co-perchings and copulations attempts). We tested both sexes separately by creating four different mixed-models. In males, we included colour morph, mass, head variables (HL, HW), and pallet quality (i.e. peak density of re-sightings at a high- or low-quality site) as fixed factors. In females we included colour morph, mass, ILL, and pallet quality as fixed factors. Regarding individual fitness, in both sexes we tested for differential reproductive and mating success using mixed-models (negative binomial distribution in males, Gaussian distribution in females) with the same predictors. We also used mixed-models to explore additional sex-specific aspects of sexual selection. In males, we estimated the average number of males with which he shared paternity of a clutch as a proxy of sperm competition intensity. We then analysed this variable with a LMM including the same predictors of the models described above. In females, we calculated fertilization success as the percentage of viable eggs from the total number of eggs laid. We tested for inter-morph differences in fertilization success using a LMM with colour morph, mass, ILL, and pallet quality as fixed factors. Capture locality and enclosure were included as random factors in every model described above. We checked that all the residuals conformed to homoscedasticity and normality assumptions, and also that they did not show over-dispersion problems in Poisson and negative binomial GLMMs (ϕ ≈ 1).

**References**

Boag, D. A. (1973) ‘Spatial relationships among members of a population of wall lizards’, *Oecologia*, 12(1), pp. 1–13. doi: 10.1007/BF00345467.

DeNardo, D. F. and Sinervo, B. (1994) ‘Effects of steroid hormone interaction on activity and home-range size of male lizards’, *Hormones and Behavior*. Academic Press, 28(3), pp. 273–287. doi: 10.1006/hbeh.1994.1023.

Fisher, R. A. (1932) *Statistical Methods for Research Workers*. 4th edn. Edited by Thomas Oliver and George Boyd. London.

Heathcote, R. J. P. *et al.* (2016) ‘Male behaviour drives assortative reproduction during the initial stage of secondary contact’, *Journal of Evolutionary Biology*. John Wiley & Sons, Ltd, 29(5), pp. 1003–1015. doi: 10.1111/jeb.12840.

Noble, D. W. A. *et al.* (2013) ‘Behavioral and morphological traits interact to promote the evolution of alternative reproductive tactics in a lizard.’, *The American naturalist*, 182(6), pp. 726–42. doi: 10.1086/673535.

Pérez i de Lanuza, G., Carazo, P. and Font, E. (2014) ‘Colours of quality: Structural (but not pigment) coloration informs about male quality in a polychromatic lizard’, *Animal Behaviour*, 90, pp. 73–81. doi: 10.1016/j.anbehav.2014.01.017.

Rose, B. (1982) ‘Lizard Home Ranges: Methodology and Functions’, *Journal of Herpetology*. Society for the Study of Amphibians and Reptiles, 16(3), p. 253. doi: 10.2307/1563718.

Sinervo, B. *et al.* (2000) ‘Testosterone, endurance, and Darwinian fitness: natural and sexual selection on the physiological bases of alternative male behaviors in side-blotched lizards’, *Hormones and Behavior*, 38(4), pp. 222–233. doi: 10.1006/hbeh.2000.1622.

Stone, P. A. and Baird, T. A. (2002) ‘Estimating Lizard Home Range: The Rose Model Revisited’, *Journal of Herpetology*. Society for the Study of Amphibians and Reptiles, 36(3), p. 427. doi: 10.2307/1566187.

Whitehead, H. (2009) ‘SOCPROG programs: Analysing animal social structures’, *Behavioral Ecology and Sociobiology*, 63(5), pp. 765–778. doi: 10.1007/s00265-008-0697-y.

**Tables**

| **Table S1.** Standardized effect size (Cohen’s *d*) calculated from published morph differences reported on different polymorphic species of lizards. | | | |
| --- | --- | --- | --- |
| **Species** | **Morph difference** | **Cohen’s *d*** | **Reference** |
| *Uta stansburiana* | Contest outcome | 1.98 | Calsbeek and Sinervo 2002 |
|  | Endurance | 2.32 | Sinervo et al. 2000 |
|  | Number of re-sightings | 1.66 | Sinervo et al. 2000 |
|  | Testosterone | 0.94 | Sinervo et al. 2000 |
|  | Home range size | 1.44 | Sinervo et al. 2000 |
|  | Number of co-sires | 0.48 | Zamudio and Sinervo 2000 |
|  | Brain cortical volume | 1.22 | LaDage et al. 2016 |
| *Urosaurus ornatus* | Mean distance between captures | 0.59 | Paterson and Blouin-Demers 2018 |
| *Liolaemus sarmientoi* | Head height | 1.14 | Fernández et al. 2018 |
|  | PC2 Aggression score | 0.51 | Fernández et al. 2018 |
|  | Field body temperature | 1.54 | Fernández et al. 2018 |
| *Ctenophorus pictus* | Fertilization success in sperm competition trials | 0.85 | Olsson et al. 2009 |
|  | Copula duration | 0.94 | Olsson et al. 2009 |
|  | Endurance | 1.01 | Tobler et al. 2012 |
|  | Reaction time against a model predator | 1.72 | Tobler et al. 2012 |
| *Ctenophorus decresii* | Aggression against a model | 0.56 | Yewers et al. 2016 |
|  | Flight initiation distance | 0.56 | Yewers et al. 2016 |
| *Podarcis melisellensis* | Bite force | 0.79 | Huyghe et al. 2009 |
|  | Corticosterone | 0.65 | Huyghe et al. 2009 |
| **Mean ± CI_95_** |  | **1.10 ± 0.24** |  |

| **Tabla S2.** Adult lizards studied in the free-ranging *P. muralis* population of Angoustrine (2006-2010). For reliable home-range estimation, we excluded lizards showing fewer than 17 re-sightings. | | | | | | |
| --- | --- | --- | --- | --- | --- | --- |
| **Sample** | **Sex** | **Colour morph** | | | | |
|  |  | o | w | y | ow | yo |
| All lizards | ♀ | 13 | 30 | 20 | 22 | 16 |
|  | ♂ | 36 | 61 | 51 | 13 | 20 |
| Residents | ♀ | 9 | 14 | 14 | 13 | 11 |
|  | ♂ | 21 | 35 | 32 | 9 | 10 |
| >17 re-sightings | ♀ | 6 | 4 | 8 | 3 | 3 |
|  | ♂ | 13 | 19 | 20 | 2 | 5 |

| **Table S3.** Measures of centrality and dispersion for spatial behaviour variables in *P. muralis* males and females from Angoustrine. | | | | | | |
| --- | --- | --- | --- | --- | --- | --- |
| **Variable** | | ** ± SEM / MED [Q1, Q3]** | | | | **Sample size** |
|  | | **♀** | | **♂** | |  |
| Resident lizards (%) | | 60.4 ± 4.9 % | | 59.1 ± 3.7 % | | 282 lizards  (101 ♀, 181 ♂) |
| **Walls visited (n)** | | **1.34 ± 0.07** | | **1.72 ± 0.06** | |  |
| Re-sightings (n) | | 8 [2, 16] | | 8 [3, 22] | |  |
| **Time elapsed (days)** | | **1.53 [2.48, 4.02]** | | **2 [1.22, 3.80]** | |  |
| Distance between sightings (m) | | 5.89 [2.99, 14.37] | | 9.60 [5.26, 14.78] | |  |
| **Linear home-range (m)** | | **13.01 [9.46, 19.52]** | | **21.71 [14.61, 29.64]** | | 83 lizards  (24 ♀, 59 ♂) |
| **Linear core-range (m)** | | **2.11 [1.39, 3.32]** | | **5.12 [3.31, 7.08]** | |  |
| **Linear exclusive range (m)** | | **0.75 [0, 2.38]** | | **4.70 [3.11, 6.67]** | |  |
| **Mean perch height (m)** | | **1.10 [0.90, 1.39]** | | **1.39 [1.11, 1.81]** | |  |
| **Spatial overlap** |  | **with ♀** | **with ♂** | **with ♀** | **with ♂** |  |
|  | **Home-core (n)** | **1.0 ± 0.2** | **2.0 ± 0.2** | **1.7 ± 0.2** | **1.5 ± 0.2** |  |
|  | **Core-core (n)** | **0.2 ± 0.1** | **1.2 ± 0.2** | **0.9 ± 0.1** | **0.3 ± 0.1** |  |
|  | Exclusive-core (n) | **-** | **0.2 ± 0.1** | **0.8 ± 0.1** | **-** |  |
| ** ± SEM** = mean ± standard error. **MED [Q1, Q3]** = median [first and third quartiles]. Bold letters mark significant sex differences (α = 0.95). | | | | | | |

| **Table S4.** Results from GLMMs examining potential differences in activity and spatial behaviour and male among colour morphs in the free-ranging population. Significant factors are highlighted in bold (α = 0.95, *P* < 0.05). Statistics for non-significant factors are included at the point of their deletion from the model. | | | | | | |
| --- | --- | --- | --- | --- | --- | --- |
| **Model** | **Variable** | **df** | ***χ^2^*** | ***P*-value** | **η_p_^2^** | **Effect size** |
| Residency (Binomial) | **SVL** | **1** | ***χ^2^* = 11.60** | ***P* < 0.001** | **0.04** | ***pseudo-r^2^* = 0.05** |
|  | Morph | 4 | *χ^2^* = 1.60 | *P* = 0.808 | 0.01 |  |
|  | Sex | 1 | *χ^2^* = 0.29 | *P* = 0.592 | 0.00 |  |
|  | Morph*Sex | 4 | *χ^2^* = 2.99 | *P* = 0.560 | 0.01 |  |
| Re-sightings (Gamma) | **SVL** | **1** | ***χ^2^* = 29.46** | ***P* < 0.001** | **0.06** | ***pseudo-r^2^* = 0.16** |
|  | Morph | 4 | *χ^2^* = 5.02 | *P* = 0.285 | 0.02 |  |
|  | Sex | 1 | *χ^2^* = 0.01 | *P* = 0.914 | 0.00 |  |
|  | Morph*Sex | 4 | *χ^2^* = 2.01 | *P* = 0.733 | 0.01 |  |
| Days elapsed (Gamma) | **SVL** | **1** | ***χ^2^* = 11.44** | ***P* < 0.001** | **0.01** | ***pseudo-r^2^* = 0.03** |
|  | Morph | 4 | *χ^2^* = 3.19 | *P* = 0.527 | 0.01 |  |
|  | **Sex** | **1** | ***χ^2^* = 4.24** | ***P* = 0.039** | **0.01** |  |
|  | Morph*Sex | 4 | *χ^2^* = 1.03 | *P* = 0.901 | 0.00 |  |
| Distance between sightings (Gamma) | **SVL** | **1** | ***χ^2^* = 6.94** | ***P* = 0.008** | **0.02** | ***pseudo-r^2^* = 0.11** |
|  | Morph | 4 | *χ^2^* = 1.32 | *P* = 0.857 | 0.00 |  |
|  | Sex | 1 | *χ^2^* = 8.65 | *P* = 0.003 | 0.00 |  |
|  | Morph*Sex | 4 | *χ^2^* = 0.46 | *P* = 0.978 | 0.00 |  |
| Linear home-range (Gaussian) | SVL | 1 | *χ^2^* = 1.30 | *P* = 0.255 | 0.02 |  |
|  | Morph | 2 | *χ^2^* = 2.20 | *P* = 0.332 | 0.02 |  |
|  | **Sex** | 1 | ***χ^2^* = 7.27** | ***P* = 0.007** | **0.13** | ***pseudo-r^2^* = 0.11** |
|  | Morph*Sex | 2 | *χ^2^* = 1.25 | *P* = 0.534 | 0.02 |  |
| Linear core-range (Gaussian) | SVL | 1 | *χ^2^* = 0.64 | *P* = 0.423 | 0.01 |  |
|  | Morph | 2 | *χ^2^* = 1.80 | *P* = 0.406 | 0.04 |  |
|  | **Sex** | 1 | ***χ^2^* = 15.12** | ***P* < 0.001** | **0.18** | ***pseudo-r^2^* = 0.20** |
|  | Morph*Sex | 2 | *χ^2^* = 4.10 | *P* = 0.128 | 0.06 |  |
| Mean perch height (Gaussian) | SVL | 1 | *χ^2^* = 3.10 | *P* = 0.080 | 0.02 |  |
|  | Morph | 2 | *χ^2^* = 1.01 | *P* = 0.603 | 0.00 |  |
|  | **Sex** | 1 | ***χ^2^* = 10.09** | ***P* = 0.001** | **0.05** | ***pseudo-r^2^* = 0.08** |
|  | Morph*Sex | 2 | *χ^2^* = 1.87 | *P* = 0.391 | 0.01 |  |
| Overlap with females (home-range) (Gamma) | SVL | 1 | *χ^2^* = 0.43 | *P* = 0.513 | 0.00 |  |
|  | Morph | 2 | *χ^2^* = 1.72 | *P* = 0.424 | 0.02 |  |
| Overlap with females (core-range) (Gamma) | SVL | 1 | *χ^2^* = 3.10 | *P* = 0.080 | 0.01 |  |
|  | Morph | 2 | *χ^2^* = 1.01 | *P* = 0.603 | 0.03 |  |
| Overlap with females (exclusive-range) (Gamma) | SVL | 1 | *χ^2^* = 0.71 | *P* = 0.400 | 0.02 |  |
|  | Morph | 2 | *χ^2^* = 2.09 | *P* = 0.351 | 0.05 |  |

| **Table S5.** Results from linear models examining potential inter-morph differences in morphometric traits in our sample of experimental lizards. Bold letters mark significant differences (α = 0.95, *P* < 0.05). | | | |
| --- | --- | --- | --- |
| **Sex** | **Trait** | **F** | **P-value** |
| Males | SVL | *F* _2, 87_ = 0.10 | *P* = 0.903 |
|  | Mass | *F* _2, 87_ = 0.07 | *P* = 0.937 |
|  | HL | *F* _2, 87_ = 0.71 | *P* = 0.493 |
|  | HW | *F* _2, 87_ = 1.25 | *P* = 0.293 |
| Females | SVL | *F* _2, 87_ = 0.39 | *P* = 0.680 |
|  | ILL | *F* _2, 87_ = 0.48 | *P* = 0.619 |
|  | **Mass** | ***F* _2, 87_ = 6.78** | ***P* = 0.002** |

| **Table S6.** Results from GLMMs examining potential differences in dominance and spatial behaviour among male colour morphs from the mesocosm experiment. Significant factors are highlighted in bold (*α* = 0.95, *P* < 0.05). Statistics for non-significant factors are included at the point of their deletion from the model. | | | | | | |
| --- | --- | --- | --- | --- | --- | --- |
| **Model** | **Variable** | **df** | **Statistic** | **P-value** | **η_p_^2^** | **Effect size ± 95%CI** |
| Social dominance (Gaussian) | Morph | 2 | ***χ^2^*** = 0.78 | *P* = 0.677 | 0.01 |  |
|  | Mass | 1 | ***χ^2^*** = 0.00 | *P* = 0.951 | 0.00 |  |
|  | HW | 1 | ***χ^2^*** = 0.12 | *P* = 0.734 | 0.00 |  |
|  | HL | 1 | ***χ^2^*** = 1.08 | *P* = 0.299 | 0.01 |  |
|  | **PQ** | **1** | ***χ^2^* = 25.56** | ***P* < 0.001** | **0.24** | ***Hedge’s g* = 1.4 [0.7, 1.7]**  ***pseudo-r^2^ =* 0.25** |
| Pallet quality (Binomial) | Morph | 2 | ***χ^2^*** = 2.13 | *P* = 0.345 | 0.03 |  |
|  | Mass | 1 | ***χ^2^*** = 0.84 | *P* = 0.360 | 0.02 |  |
|  | HW | 1 | ***χ^2^*** = 1.68 | *P* = 0.196 | 0.03 |  |
|  | HL | 1 | ***χ^2^*** = 0.25 | *P* = 0.619 | 0.00 |  |
|  | **Dominance** | **1** | ***χ^2^* = 23.97** | ***P* < 0.001** | **0.25** | ***OR =* 4.5 [2.3, 11.7]**  ***pseudo-r^2^ =* 0.30** |
| Home-range size (Gaussian) | Morph | 2 | ***χ^2^*** = 2.14 | *P* = 0.344 | 0.03 |  |
|  | Mass | 1 | ***χ^2^*** = 2.01 | *P* = 0.157 | 0.03 |  |
|  | HW | 1 | ***χ^2^*** = 0.96 | *P* = 0.327 | 0.01 |  |
|  | HL | 1 | ***χ^2^*** = 0.31 | *P* = 0.580 | 0.00 |  |
|  | **Dominance** | **1** | ***χ^2^* = 23.97** | ***P* < 0.001** | **0.36** | ***pseudo-r^2^ =* 0.30** |
| Core-range size (Gaussian) | Morph | 2 | ***χ^2^*** = 3.18 | *P* = 0.204 | 0.04 |  |
|  | Mass | 1 | ***χ^2^*** = 0.03 | *P* = 0.863 | 0.00 |  |
|  | HW | 1 | ***χ^2^*** = 0.35 | *P* = 0.552 | 0.00 |  |
|  | HL | 1 | ***χ^2^*** = 0.43 | *P* = 0.512 | 0.01 |  |
|  | **Dominance** | **1** | ***χ^2^* = 31.90** | ***P* < 0.001** | **0.35** | ***pseudo-r^2^ =* 0.31** |
| Overlap with females (k50) (Poisson) | Morph | 2 | ***χ^2^*** = 1.46 | *P* = 0.481 | 0.01 |  |
|  | Mass | 1 | ***χ^2^*** = 0.02 | *P* = 0.898 | 0.00 |  |
|  | HW | 1 | ***χ^2^*** = 0.00 | *P* = 0.972 | 0.00 |  |
|  | HL | 1 | ***χ^2^*** = 0.13 | *P* = 0.715 | 0.00 |  |
|  | **PQ** | **1** | ***χ^2^* = 31.90** | ***P* < 0.001** | **0.28** | **HQ-LQ = 3.0 ± 1.2 females**  ***pseudo-r^2^ =* 0.24** |

| **Table S7.** Results from GLMMs examining potential differences in spatial behaviour among female colour morphs from the mesocosm experiment. Significant factors are highlighted in bold (*α* = 0.95, *P* < 0.05). Statistics for non-significant factors are included at the point of their deletion from the model. | | | | | | |
| --- | --- | --- | --- | --- | --- | --- |
| **Model** | **Variable** | **df** | **Statistic** | **P-value** | **η_p_^2^** | **Effect size ± 95%CI** |
| Pallet quality (Binomial) | Morph | 2 | ***χ^2^*** = 2.95 | *P* = 0.229 | 0.05 |  |
|  | Mass | 1 | ***χ^2^*** = 3.75 | *P* = 0.206 | 0.02 |  |
|  | ILL | 1 | ***χ^2^*** = 0.19 | *P* = 0.662 | 0.00 |  |
| Home-range size (Gaussian) | **Morph** | **2** | ***χ^2^* = 15.68** | ***P* < 0.001** | **0.18** | **W-O = 6.4 ± 3.5 m^2^**  ***pseudo-r^2^ =* 0.18** |
|  | Mass | 1 | ***χ^2^*** = 0.06 | *P* = 0.802 | 0.03 |  |
|  | ILL | **1** | ***χ^2^* = 3.90** | ***P* = 0.048** | **0.05** |  |
| Core-range size (Gaussian) | **Morph** | **2** | ***χ^2^* = 15.54** | ***P* < 0.001** | **0.19** | **W-O = 1.7 ± 0.9 m^2^**  ***pseudo-r^2^ =* 0.16** |
|  | Mass | 1 | ***χ^2^*** = 0.03 | *P* = 0.866 | 0.00 |  |
|  | ILL | 1 | ***χ^2^*** = 1.82 | *P* = 0.178 | 0.01 |  |
| Overlap with males (k50) (Poisson) | Morph | 2 | ***χ^2^*** = 1.31 | *P* = 0.520 | 0.03 |  |
|  | Mass | 1 | ***χ^2^*** = 0.02 | *P* = 0.879 | 0.00 |  |
|  | ILL | 1 | ***χ^2^*** = 1.20 | *P* = 0.273 | 0.02 |  |
|  | **PQ** | **1** | ***χ^2^* = 8.42** | ***P* = 0.004** | **0.13** | **LQ-HQ = 1.7 ± 1.0 males**  ***pseudo-r^2^ =* 0.08** |

| **Table S8**. Male-male competitive interactions recorded within the experimental enclosures, sorted by morph combination and contest outcome. | | | | | |
| --- | --- | --- | --- | --- | --- |
|  |  | Loser morph | | |  |
|  |  | Orange | White | Yellow | Total (heteromorphic) |
| Winner morph | Orange | 34 (14) | 57 (26) | 85 (32) | 142 (58) |
|  | White | 97 (25) | 44 (14) | 74 (28) | 171 (53) |
|  | Yellow | 52 (22) | 58 (22) | 42 (17) | 110 (44) |
|  | Total (heteromorphic) | 149 (47) | 115 (48) | 159 (60) | 423 (155) |
| Plain numbers = absolute frequency. In brackets = absolute frequency after dealing with pseudo-replication. | | | | | |

| **Table S9.** Results from GLMMs examining potential differences in sexual behaviour and fitness among male colour morphs from the mesocosm experiment. Significant factors are highlighted in bold (*α* = 0.95, *P* < 0.05). Statistics for non-significant factors are included at the point of their deletion from the model. | | | | | | |
| --- | --- | --- | --- | --- | --- | --- |
| **Model** | **Variable** | **df** | **Statistic** | **P-value** | **η_p_^2^** | **Effect size ± 95%CI** |
| Females encountered in reproductive interactions (n) (NegBin) | Morph | 2 | ***χ^2^*** = 0.09 | *P* = 0.958 | 0.00 |  |
|  | Mass | 1 | ***χ^2^*** = 0.00 | *P* = 0.970 | 0.00 |  |
|  | HW | 1 | ***χ^2^*** = 0.12 | *P* = 0.967 | 0.00 |  |
|  | HL | 1 | ***χ^2^*** = 0.03 | *P* = 0.858 | 0.00 |  |
|  | **PQ** | **1** | ***χ^2^* = 27.24** | ***P* < 0.001** | **0.30** | **HQ-LQ = 2.7 ± 0.9 females**  ***pseudo-r^2^ =* 0.36** |
| Females in co-perching (n) (NegBin) | Morph | 2 | ***χ^2^*** = 0.49 | *P* = 0.784 | 0.00 |  |
|  | Mass | 1 | ***χ^2^*** = 2.26 | *P* = 0.133 | 0.02 |  |
|  | HW | 1 | ***χ^2^*** = 0.02 | *P* = 0.891 | 0.00 |  |
|  | HL | 1 | ***χ^2^*** = 1.76 | *P* = 0.185 | 0.01 |  |
|  | **PQ** | **1** | ***χ^2^* = 29.73** | ***P* < 0.001** | **0.32** | **HQ-LQ = 3.0 ± 1.5 females**  ***pseudo-r^2^ =* 0.32** |
| Females in copulatory behaviour (n) (NegBin) | Morph | 2 | ***χ^2^*** = 1.14 | *P* = 0.566 | 0.01 |  |
|  | Mass | 1 | ***χ^2^*** = 0.00 | *P* = 0.968 | 0.00 |  |
|  | HW | 1 | ***χ^2^*** = 2.01 | *P* = 0.156 | 0.01 |  |
|  | HL | 1 | ***χ^2^*** = 0.02 | *P* = 0.900 | 0.00 |  |
|  | **PQ** | **1** | ***χ^2^* = 13.36** | ***P* < 0.001** | **0.13** | **HQ-LQ = 1.1 ± 0.7 females**  ***pseudo-r^2^ =* 0.13** |
| Relative mating success (NegBin) | Morph | 2 | ***χ^2^*** = 3.32 | *P* = 0.190 | 0.04 |  |
|  | Mass | 1 | ***χ^2^*** = 0.10 | *P* = 0.751 | 0.00 |  |
|  | HW | 1 | ***χ^2^*** = 0.14 | *P* = 0.707 | 0.00 |  |
|  | HL | 1 | ***χ^2^*** = 1.20 | *P* = 0.274 | 0.01 |  |
|  | PQ | 1 | *χ^2^* = 2.60 | *P* = 0.107 | 0.03 |  |
| Relative reproductive success (NegBin) | Morph | 2 | ***χ^2^*** = 2.63 | *P* = 0.268 | 0.02 |  |
|  | Mass | 1 | ***χ^2^*** = 0.00 | *P* = 0.971 | 0.00 |  |
|  | HW | 1 | ***χ^2^*** = 0.00 | *P* = 0.960 | 0.00 |  |
|  | HL | 1 | ***χ^2^*** = 1.91 | *P* = 0.167 | 0.01 |  |
|  | **PQ** | **1** | ***χ^2^* = 11.28** | ***P* < 0.001** | **0.12** | **HQ-LQ = 0.8 ± 0.4**  ***pseudo-r^2^ =* 0.13** |
| Sperm competition intensity (Gaussian) | Morph | 2 | ***χ^2^*** = 1.15 | *P* = 0.562 | 0.02 |  |
|  | Mass | 1 | ***χ^2^*** = 2.11 | *P* = 0.147 | 0.02 |  |
|  | HW | 1 | ***χ^2^*** = 1.14 | *P* = 0.285 | 0.03 |  |
|  | HL | 1 | ***χ^2^*** = 0.02 | *P* = 0.875 | 0.02 |  |
|  | **PQ** | **1** | ***χ^2^* = 10.60** | ***P* = 0.001** | **0.19** | **HQ-LQ = 0.8 ± 0.5 males**  ***pseudo-r^2^ =* 0.15** |

| **Table S10**. Shared paternity associations among males of different colour morphs during the enclosure experiment. | | | | | | | |
| --- | --- | --- | --- | --- | --- | --- | --- |
|  | O-O | W-W | Y-Y | O-W | Y-O | W-Y | Total |
| Observed | 4 | 4 | 7 | 13 | 16 | 20 | 64 |
| Expected | 5.33 | 5.33 | 5.33 | 16 | 16 | 16 | 64 |
| Probability | 0.083 | 0.083 | 0.083 | 0.25 | 0.25 | 0.25 |  |

| **Table S11.** Results from GLMMs examining potential differences in sexual behaviour and fitness among female colour morphs from the mesocosm experiment. Significant factors are highlighted in bold (α = 0.95, *P* < 0.05). Statistics for non-significant factors are included at the point of their deletion from the model. | | | | | | |
| --- | --- | --- | --- | --- | --- | --- |
| **Model** | **Variable** | **df** | **Statistic** | **P-value** | **η_p_^2^** | **Effect size ± 95%CI** |
| Males encountered in reproductive interactions (n) (Gaussian) | Morph | 2 | ***χ^2^*** = 0.31 | *P* = 0.856 | 0.00 |  |
|  | Mass | 1 | ***χ^2^*** = 3.69 | *P* = 0.055 | 0.07 |  |
|  | ILL | 1 | ***χ^2^*** = 1.99 | *P* = 0.158 | 0.03 |  |
|  | **PQ** | **1** | ***χ^2^* = 5.59** | ***P* = 0.018** | **0.08** | **LQ-HQ = 1.1. ± 0.8 males**  ***pseudo-r^2^ =* 0.06** |
| Eggs produced (n) (Gaussian) | Morph | 2 | ***χ^2^*** = 4.61 | *P* = 0.110 | 0.07 |  |
|  | **Mass** | **1** | ***χ^2^* = 14.35** | ***P* < 0.001** | **0.19** | ***pseudo-r^2^ =* 0.18** |
|  | ILL | 1 | ***χ^2^*** = 0.59 | *P* = 0.444 | 0.01 |  |
|  | PQ | 1 | *χ^2^* = 0.06 | *P* = 0.800 | 0.00 |  |
| Fertilization success (%) (Gaussian) | Morph | 2 | ***χ^2^*** = 2.18 | *P* = 0.335 | 0.04 |  |
|  | Mass | 1 | ***χ^2^*** = 0.58 | *P* = 0.447 | 0.01 |  |
|  | ILL | 1 | ***χ^2^*** = 0.08 | *P* = 0.784 | 0.00 |  |
|  | **PQ** | **1** | ***χ^2^* = 4.13** | ***P* = 0.042** | **0.07** | **LQ-HQ = 0.29 ± 0.27 %**  ***pseudo-r^2^ =* 0.07** |
| Laying date (n) (Gaussian) | Morph | 2 | ***χ^2^*** = 0.69 | *P* = 0.709 | 0.01 |  |
|  | **Mass** | **1** | ***χ^2^* = 6.03** | ***P* = 0.014** | **0.06** | ***pseudo-r^2^ =* 0.11** |
|  | **ILL** | **1** | ***χ^2^* = 6.03** | ***P* = 0.014** | **0.00** |  |
|  | PQ | 1 | *χ^2^* = 0.02 | *P* = 0.878 | 0.07 |  |
| Relative mating success (NegBin) | Morph | 2 | ***χ^2^*** = 3.24 | *P* = 0.197 | 0.05 |  |
|  | Mass | 1 | ***χ^2^*** = 0.90 | *P* = 0.898 | 0.01 |  |
|  | ILL | 1 | ***χ^2^*** = 1.57 | *P* = 0.210 | 0.04 |  |
|  | **PQ** | **1** | ***χ^2^* = 4.50** | ***P* = 0.034** | **0.04** | **LQ-HQ = 0.7 ± 0.6**  ***pseudo-r^2^ =* 0.07** |
| Relative reproductive success (NegBin) | Morph | 2 | ***χ^2^*** = 3.62 | *P* = 0.164 | 0.04 |  |
|  | Mass | 1 | ***χ^2^*** = 0.00 | *P* = 0.964 | 0.00 |  |
|  | ILL | 1 | ***χ^2^*** = 0.97 | *P* = 0.326 | 0.01 |  |
|  | PQ | 1 | *χ^2^* = 0.90 | *P* = 0.343 | 0.01 |  |

**Figures**

**
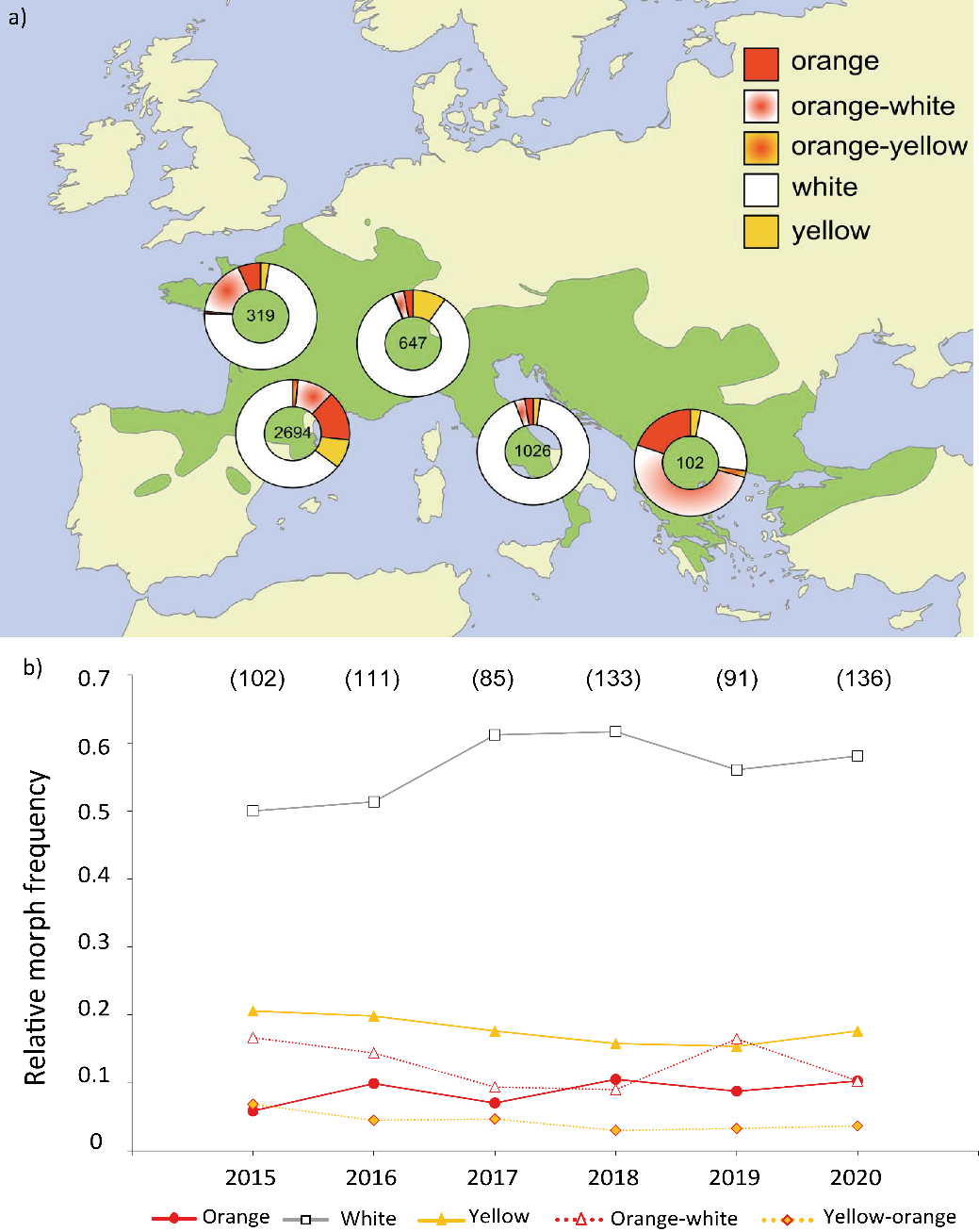
**

**Figure S1.** a) Native distribution of *Podarcis muralis* (green shading) with pie charts representing the natural morph frequencies pooled by geographically distant sub-lineages. White morph lizards are usually the most common. The number of lizards sampled is indicated inside the pie charts. Figure extracted from Andrade *et al.* (2018) and reproduced here with permission from the authors. b) Variation in morph relative frequencies (pooling males and females) for a six-year period (2015-2020) in the study population of Angoustrine. Sample size for each year is provided in brackets**.**

**
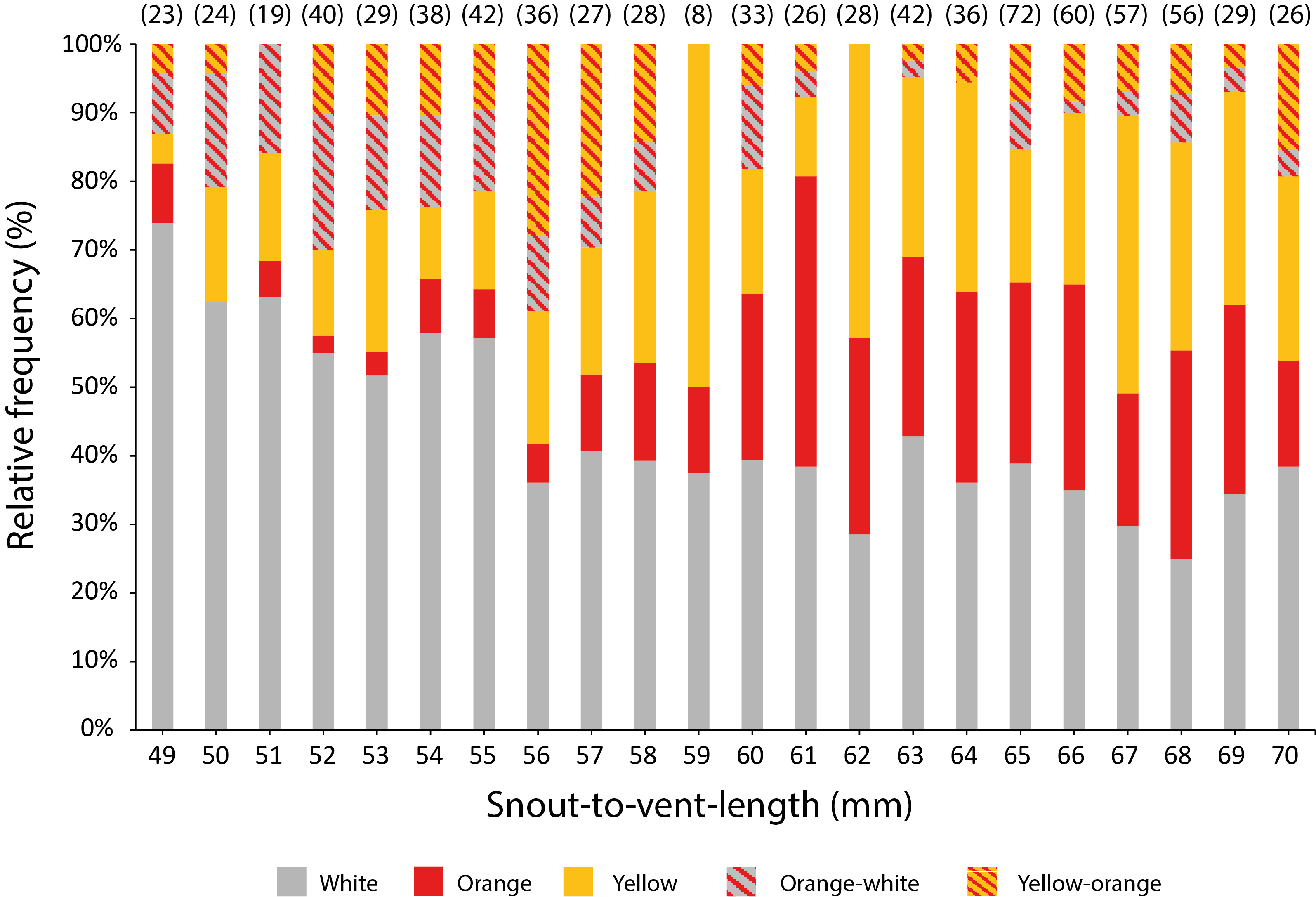
**

**Figure S2.** Colour morph relative frequencies in males from Angoustrine showing different body sizes (snout-to-vent-length, SVL). Sample size for each SVL are provided in brackets. White morph frequency falls steeply toward larger body sizes with the largest change in slope occurring between 55 and 56 mm, after which colour morph frequencies tend to stabilize (as suggested by results in Pérez i de Lanuza et al., 2013). Given this result, we consider 56 mm a suitable cut-off value of SVL for reliable morph determination in the localities sampled. This cut-off, however, could vary among different populations.


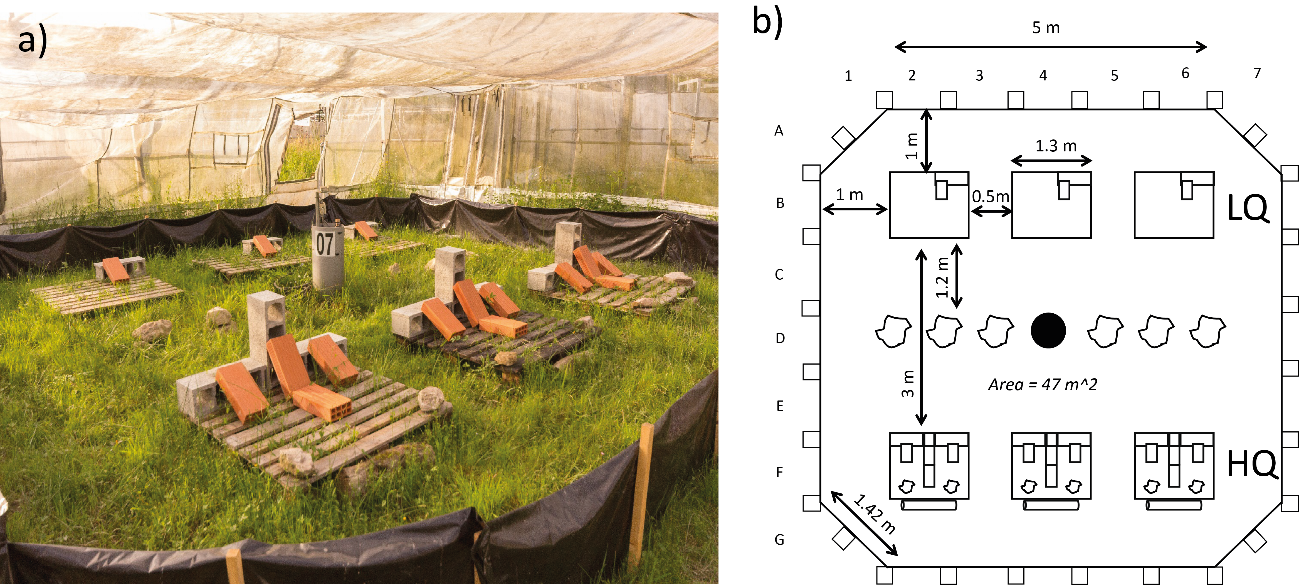


**Figure S3.** a) Photography of one of the experimental enclosures at the Metatron research facility (CNRS; Caumont, France) associated to the Station d’Ecologie Theóretique et Experimentale (SETE, Moulis, France). b) Diagram of the spatial arrangement inside the experimental enclosures (HQ = high-quality sites, LQ = low-quality sites).


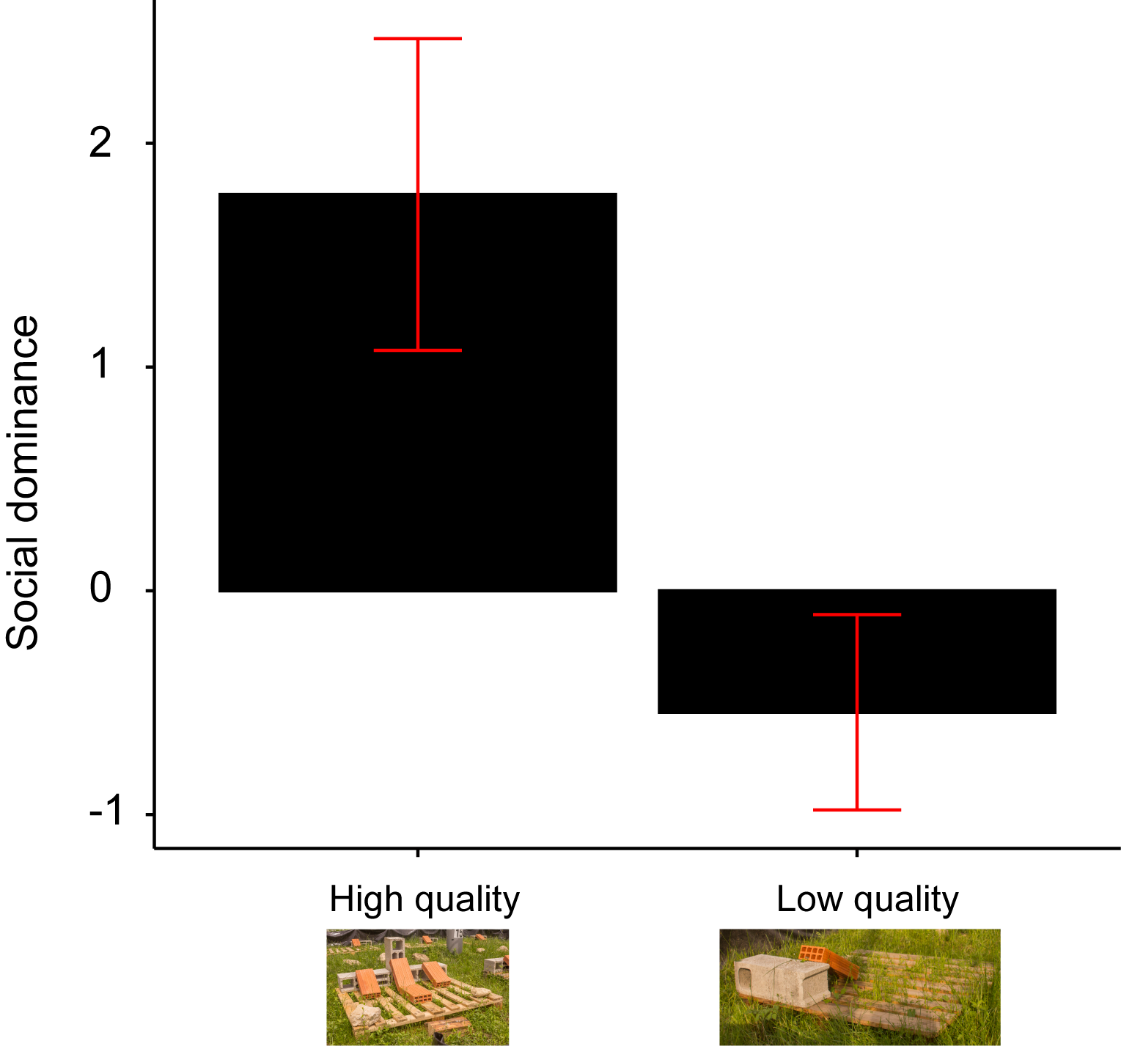


**Figure S4.** Barplot showing the difference in social dominance between males occupying high- and low-quality sites. Red bars represent 95% CI.


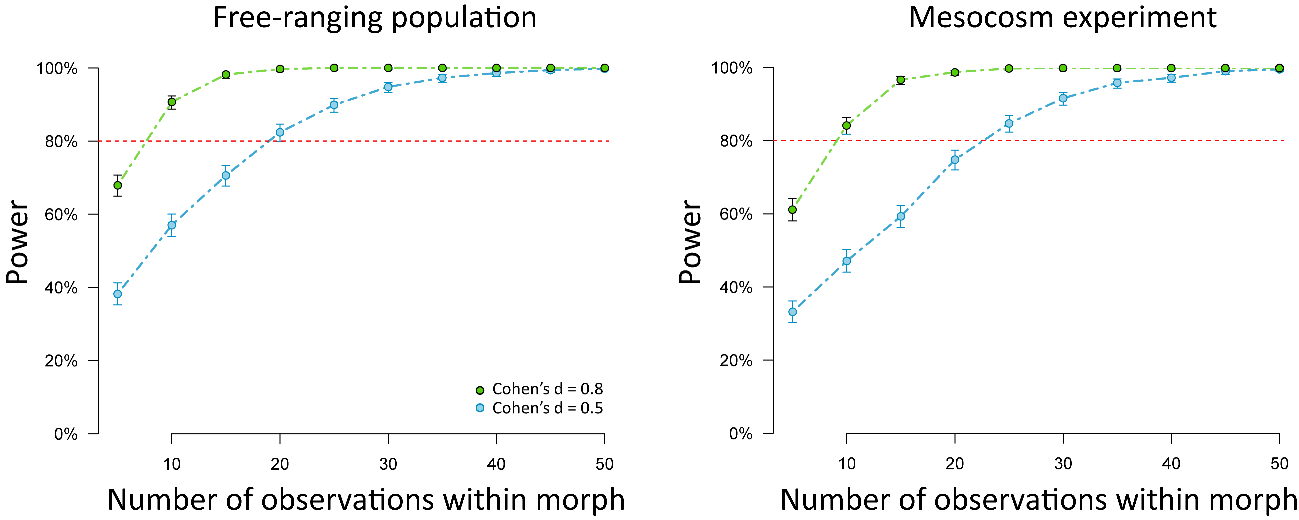


**Figure S5.** Power (± CI_95_) to detect a medium-sized (Cohen’s *d* = 0.5, blue line) or large effect size (Cohen’s *d* = 0.8, green line) for morph differences in the free-ranging population (left) or the mesocosm experiment (right), calculated over a range of sample sizes (5-50) using the powerCurve function in the *simr* package (1000 simulations). Estimates for fixed and random effects (besides morph) replicate those obtained in the LMMs exploring male morph differences in home-range size (left) or social dominance (right). The curves confirm that our mixed-model statistical designs (with 13-30 lizards per morph) were sufficiently powered to detect biologically relevant morph differences in socio-sexual behaviour.

**Video recordings**


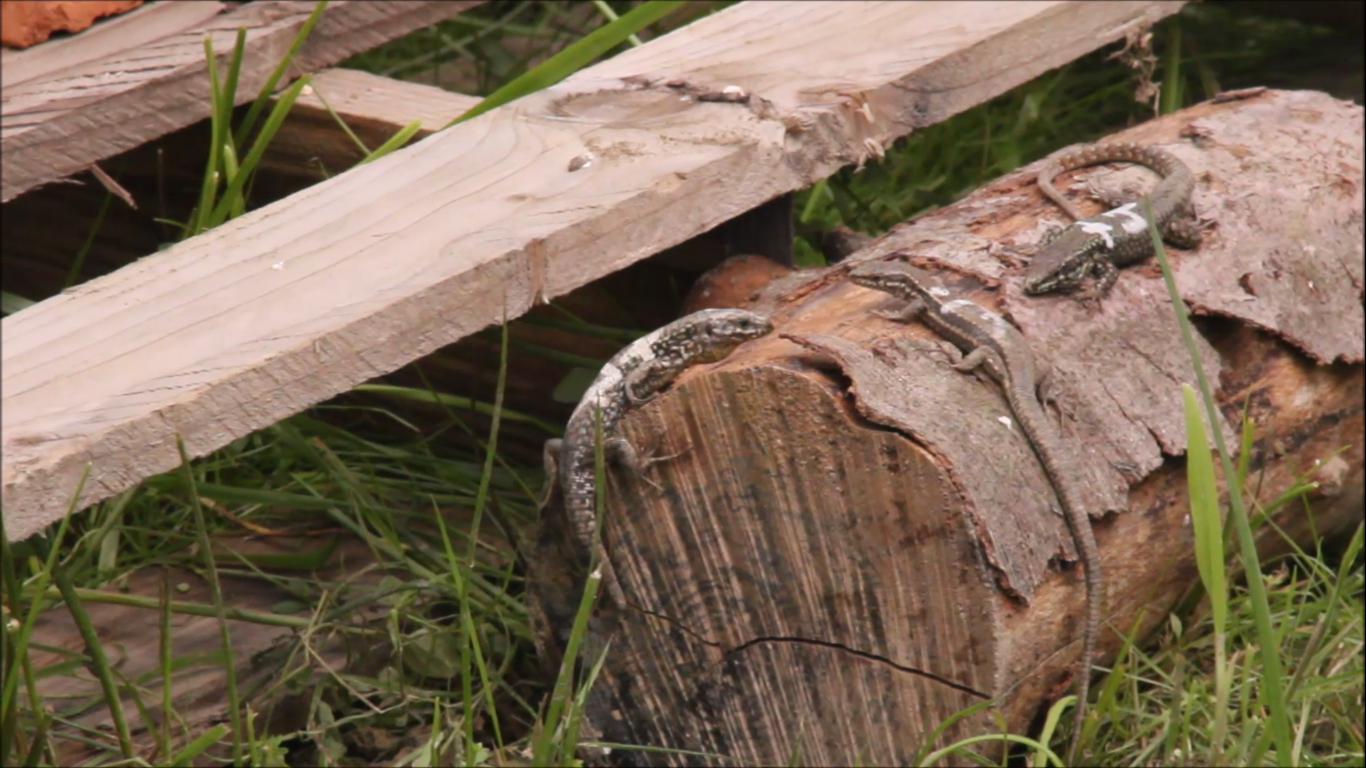


**Video S1.** Screenshot of a social interaction observed during the period of behavioural observations within the experimental enclosures. The full recording can be downloaded as a separate MP4 file.
